# Distinct temporal roles for the promyelocytic leukaemia (PML) protein in the sequential regulation of intracellular host immunity to HSV-1 infection

**DOI:** 10.1371/journal.ppat.1006769

**Published:** 2018-01-08

**Authors:** Thamir Alandijany, Ashley P. E. Roberts, Kristen L. Conn, Colin Loney, Steven McFarlane, Anne Orr, Chris Boutell

**Affiliations:** 1 MRC-University of Glasgow Centre for Virus Research (CVR), Garscube Campus, Glasgow, Scotland, United Kingdom; 2 Department of Medical Laboratory Technology, Faculty of Applied Medical Sciences, King Abdulaziz University, Jeddah, Saudi Arabia; 3 Department of Biochemistry, University of Alberta, Edmonton, AB, Canada; Louisiana State University Health Sciences Center, UNITED STATES

## Abstract

Detection of viral nucleic acids plays a critical role in the induction of intracellular host immune defences. However, the temporal recruitment of immune regulators to infecting viral genomes remains poorly defined due to the technical difficulties associated with low genome copy-number detection. Here we utilize 5-Ethynyl-2’-deoxyuridine (EdU) labelling of herpes simplex virus 1 (HSV-1) DNA in combination with click chemistry to examine the sequential recruitment of host immune regulators to infecting viral genomes under low multiplicity of infection conditions. Following viral genome entry into the nucleus, PML-nuclear bodies (PML-NBs) rapidly entrapped viral DNA (vDNA) leading to a block in viral replication in the absence of the viral PML-NB antagonist ICP0. This pre-existing intrinsic host defence to infection occurred independently of the vDNA pathogen sensor IFI16 (Interferon Gamma Inducible Protein 16) and the induction of interferon stimulated gene (ISG) expression, demonstrating that vDNA entry into the nucleus alone is not sufficient to induce a robust innate immune response. Saturation of this pre-existing intrinsic host defence during HSV-1 ICP0-null mutant infection led to the stable recruitment of PML and IFI16 into vDNA complexes associated with ICP4, and led to the induction of ISG expression. This induced innate immune response occurred in a PML-, IFI16-, and Janus-Associated Kinase (JAK)-dependent manner and was restricted by phosphonoacetic acid, demonstrating that vDNA polymerase activity is required for the robust induction of ISG expression during HSV-1 infection. Our data identifies dual roles for PML in the sequential regulation of intrinsic and innate immunity to HSV-1 infection that are dependent on viral genome delivery to the nucleus and the onset of vDNA replication, respectively. These intracellular host defences are counteracted by ICP0, which targets PML for degradation from the outset of nuclear infection to promote vDNA release from PML-NBs and the onset of HSV-1 lytic replication.

## Author summary

Intrinsic and innate immunity act to restrict the replication of many clinically important viral pathogens. However, the temporal regulation of these two arms of host immunity during virus infection remains poorly defined. A key aspect in the regulation of these intracellular immune defences during herpesvirus infection is the rapid recruitment of constitutively expressed immune regulators to infecting viral genomes. Here we show that at physiologically low multiplicities of infection, PML-NBs rapidly entrap HSV-1 genomes upon nuclear entry. Saturation of this pre-existing intrinsic host defence led to the stable recruitment of the vDNA pathogen sensor IFI16 to HSV-1 vDNA and the induction of ISG expression, an induced innate immune response dependent on the initiation of vDNA replication. Importantly, both intrinsic and innate arms of host immunity required PML, the principle scaffolding protein of PML-NBs. Our research identifies dual roles for PML in the sequential regulation of intracellular host immunity during HSV-1 infection, and highlights distinct phases in host immune factor recruitment to infecting viral genomes required for the temporal regulation of intracellular host immune defences during herpesvirus infection.

## Introduction

Intrinsic, innate, and adaptive arms of host immunity cooperatively supress the replication and spread of invading viral pathogens. Conferred by constitutively expressed host-cell restriction factors, intrinsic immunity is the first line of intracellular defence against infection (reviewed in [1–3]). In contrast, innate immune defences are upregulated following the activation of Pattern Recognition Receptors (PRRs) that detect Pathogen-Associated Molecular Patterns (PAMPs) unique to microbial pathogens, including foreign viral nucleic acids. PRR activation induces downstream signalling events that culminate in the expression of antiviral host genes, principally cytokines (including interferons) and interferon stimulated gene (ISG) products (reviewed in [4–6]). This induced innate immune response confers a broadly refractory antiviral state that limits virus propagation and stimulates adaptive immune responses. Consequently, many viruses have evolved counter measures to antagonize intrinsic and innate immune defences to promote their efficient propagation and transmission to new hosts.

A key event in the regulation of intracellular immune defences during herpesvirus infection is the rapid recruitment of constitutively expressed host factors to sites in close proximity to infecting viral genomes upon nuclear entry (reviewed in [1, 7]). These factors include core constituent proteins of Promyelocytic Leukaemia Nuclear Bodies (PML-NBs; notably PML, Sp100, Daxx and ATRX; [8–10]), innate immune regulators (IFI16, cGAS, and STING; [11–14]), DNA Damage Response (DDR) proteins (γH2AX, Mdc1, 53BP1, and BRCA1; [15]), and core component proteins of the SUMOylation pathway (SUMO-1, SUMO-2/3, PIAS1, and PIAS4 [16–19]). The recruitment of these host factors represents the earliest detectable nuclear responses to infection, and have been linked to the repression of viral gene expression and PRR activation in the regulation of intrinsic and innate immune defences, respectively. The importance of PML-NB constituent proteins and IFI16 in the regulation of intracellular immunity is highlighted by the fact that many viruses have evolved strategies to antagonise these key immune regulators (reviewed in [1, 4, 6, 20, 21]). One of the first viral proteins to be expressed during Herpes Simplex Virus 1 (HSV-1) infection is ICP0, a viral RING-finger ubiquitin ligase that promotes the degradation and dispersal of host factors, including PML and IFI16 [11, 22–31], away from infecting viral genomes (reviewed in [7, 32]). This activity inhibits viral genome silencing and the induction of ISG expression, thereby promoting the efficient onset of HSV-1 gene expression and replication. Viral mutants that do not express ICP0, or carry mutations that impair its ubiquitin ligase activity, are highly susceptible to host-cell restriction at low multiplicities of infection (MOI) and are hypersensitive to interferon (IFN) treatment [33–38]. The use of such mutants has been critical in defining many aspects relating to the regulation of intrinsic and innate immunity during herpesvirus infection. Studies analysing the recruitment of host immune regulators to infecting viral genomes have typically relied on high MOI conditions due to the technical challenges associated with low genome copy-number detection. A defining hallmark of intrinsic immunity, however, is that this host defence is readily saturated under high MOI conditions due to limiting levels of pre-existing host factors. Thus, much of the mechanistic detail of immune regulator recruitment to infecting HSV-1 genomes has been established using viral mutants at input genome levels that saturate intrinsic host defences. Consequently, the temporal recruitment of intrinsic and innate immune regulators to infecting viral genomes remains poorly defined, specifically under low MOI conditions pertinent to wild-type (WT) herpesvirus infections observed in a clinical setting.

Here we use fluorophore conjugation by click chemistry to investigate the temporal recruitment of intrinsic and innate immune regulators to 5-Ethynyl-2’-deoxyuridine (EdU) labelled HSV-1 genomes under physiologically low MOI conditions (0.1 to 3 PFU/cell). HSV-1 genomes were readily detected in the nucleus within 30 minutes of infection (post-addition of virus) and to be stably entrapped within PML-NBs in restrictive cell types prior to PML-NB disruption and genome release by ICP0. PML-NB entrapment of vDNA occurred independently of the PRR sensor IFI16 and ISG expression, demonstrating that this intrinsic host response does not directly contribute to the induction of innate immunity. Saturation of this host defence during HSV-1 ICP0-null mutant infection led to the stable recruitment of PML and IFI16 into vDNA complexes associated with ICP4, and the subsequent induction of ISGs. This induced innate immune response occurred in a PML-, IFI16-, and Janus associated kinase (JAK) dependent manner, which could be suppressed by the vDNA polymerase inhibitor phosphonoacetic acid (PAA). These data demonstrate that vDNA entry into the nucleus alone under low MOI conditions is not sufficient to stimulate a robust innate immune response to HSV-1 nuclear infection, which only occurs after the onset of vDNA replication. We show that intrinsic and innate arms of intracellular host immunity act sequentially, as inhibition of innate immune signalling could not relieve the intrinsic cellular restriction of an HSV-1 ICP0-null mutant, but instead led to significantly enhanced virus yields under infection conditions that enabled the onset of vDNA replication. Collectively, our data demonstrate that intrinsic and innate arms of host immunity are temporally distinct immune events activated in response to vDNA nuclear entry and the onset of vDNA replication, respectively. Our data identifies distinct roles for PML in the sequential regulation of these intracellular immune defences to HSV-1 infection, findings that are likely to be highly pertinent in the cellular restriction of many nuclear replicating viral pathogens.

## Results

### Direct visualization of bio-orthogonally labelled input HSV-1 genomes at low MOI

Microscopy studies have played pivotal roles in the identification of host factors and signalling pathways that contribute to the intracellular regulation of intrinsic and innate immunity during herpesvirus infection (reviewed in [1, 4, 7, 32, 39]). However, the detection of viral genomes under low MOI conditions, which do not saturate intrinsic host defences, remains a significant technical challenge. To date, viral genome localization studies have relied on the indirect detection of vDNA through immunolabelling or fluorescent tagging of vDNA binding proteins, for example the viral immediate early (IE) transcription factor ICP4 or the early (E) single-stranded vDNA binding protein ICP8 [8–10, 13, 14, 40]. The use of vDNA binding proteins limits temporal resolution of host factor recruitment to infecting viral genomes, as genome detection requires the successful expression of viral gene products that may compete with, or displace, host factors bound to vDNA. Consequently, this strategy is suboptimal for the examination of early intrinsic host immune defences that influence the cellular restriction of viral gene expression. While direct vDNA labelling strategies have been employed, most notably fluorescent *in situ* hybridization (FISH; [8, 10]), such approaches require harsh denaturing conditions which impair host antigen detection ([41], personal communication J. Brown), and have not been widely adopted. Recent advances in direct bio-orthogonal nucleic acid labelling, using Ethynyl-tagged deoxynucleotides in combination with fluorescent labelling by click chemistry techniques, have enabled the direct visualization of vDNA during both Adeno- and Herpesvirus infection ([42–46]). We sought to apply this technique by purifying either EdU or EdC labelled HSV-1 virions (HSV-1^EdU^ or HSV-1^EdC^, respectively) and infecting cells at low MOI (≤ 3 PFU/cell) to examine the temporal recruitment of intrinsic and innate immune regulators to input viral genomes following nuclear entry.

**Fig 1:**
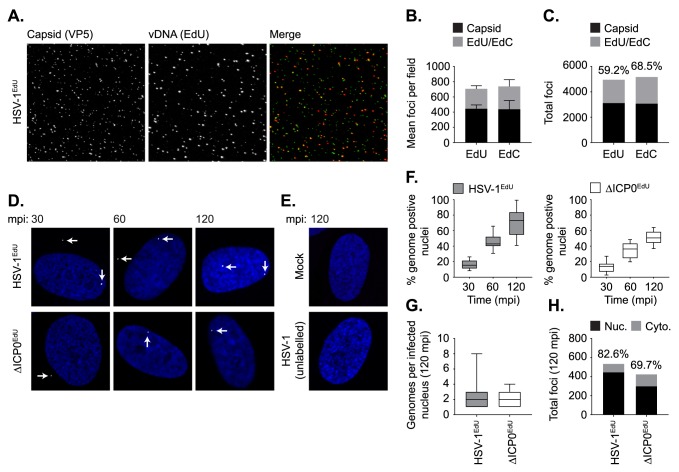
Direct visualization of bio-orthogonally labelled HSV-1 genomes. Quantitation of bio-orthogonally labelled vDNA in HSV-1 virions. HSV-1^EdU^ or HSV-1^EdC^ virions were subjected to partial denaturation with 2M GuHCl at 4°C for 60 mins to release vDNA [47]. vDNA (red) was detected by click chemistry and capsid (green) by indirect immunofluorescence staining. (A) Representative confocal images showing vDNA release and expansion from partially denatured HSV-1^EdU^ virions following GuHCl treatment. (B,C) Stack plots showing the relative population of EdU or EdC positive vDNA labelled HSV-1 virions. n ≥ 3000 particles per population derived from 3 independent experiments. Minimum estimates for viral genome labelling efficiency are shown (%). (D,E) HFt cells were mock or HSV-1 infected with either unlabelled or EdU labelled WT (HSV-1^EdU^) or ICP0-null mutant HSV-1 (ΔICP0^EdU^) at an MOI of 3 PFU/cell. Cells were fixed and permeabilized at the indicated times minutes post-infection (mpi; post-addition of virus). vDNA was detected by click chemistry (white arrows) and nuclei stained with DAPI (blue). Representative confocal images showing the nuclear accumulation of HSV-1^EdU^ and ΔICP0^EdU^ genomes over time (30–120 mpi; as indicated), with genome detection specific to input EdU labelled virus. (F) Quantitation of HSV-1^EdU^ or ΔICP0^EdU^ genome positive nuclei over time (as in D). Boxes: 25^th^ to 75^th^ percentile range; black line: median; whiskers: 5^th^ to 95^th^ percentile range. n ≥ 300 cells per sample population derived from a minimum of 3 independent experiments. (G) Number of genomes detected within infected nuclei at 120 mpi. Boxes: 25^th^ to 75^th^ percentile range; black line: median; whiskers: minimum and maximum range of sample. n ≥ 200 cells per sample population derived from a minimum of 3 independent experiments. (H) Nuclear (Nuc.) and cytoplasmic (Cyto.) distribution of genome foci detected at 120 mpi. n ≥ 300 cells per sample population derived from a minimum of 3 independent experiments. Percentage of total genomes detected within the nucleus is shown (%).

Successful labelling of vDNA and the purification of high titre WT or ICP0-null mutant HSV-1^EdU^ or HSV-1^EdC^ stocks was achieved by infecting Retinal Pigmented Epithelial (RPE) cells (see Materials and methods; S1A–S1C Fig). Notably, many laboratory cell lines were unable to support efficient viral propagation at nucleotide concentrations exceeding 1 μM in an Ethynyl-tag dependent manner (S1 Table, S1D–S1F Fig). As RPE cells were restrictive to HSV-1 ICP0-null mutant replication (see below) and sensitive to Ethynyl-tagged deoxynucleotide labelling in the absence of ICP0 (S1G–S1I Fig), we selected the lowest dose of 0.5 μM EdU or EdC for genome labelling to enable comparative recruitment studies to input viral genomes in the presence or absence of ICP0. *In vitro* genome release assays demonstrated that ≥ 60% of virions contained EdU or EdC labelled viral genomes detectable by click chemistry following partial denaturation of the HSV-1 capsid by 2M guanidine hydrochloride (GuHCl, Fig 1A–1C, S2 Fig; [47]). Particle to plaque forming unit (PFU) analysis of virus preparations grown in the presence of EdU demonstrated that HSV-1^EdU^ labelled virions had a roughly equivalent ratio to that of unlabelled control virus preparations (within 3-fold; S2 Table). These data demonstrate that EdU labelling of vDNA was not significantly detrimental to virion production or infectivity under these labelling conditions.

A time course of infection of human foreskin fibroblast (HFt) cells with WT (HSV-1^EdU^) or ICP0-null (ΔICP0^EdU^) mutant HSV-1 demonstrated that vDNA could be readily detected within the nuclei of infected cells as early as 30 minutes post-infection (mpi; post-addition of virus), with > 70% of nuclei containing at least 1 (median average of 2) HSV-1^EdU^ genome foci by 120 mpi (Fig 1D, 1F and 1G). Signal detection was dependent on both HSV-1 infection and EdU vDNA labelling, demonstrating that fluorescent click signal(s) were specific to input EdU labelled vDNA (Fig 1E), with the majority of genome signals (≥ 70%) observed within the nucleus (Fig 1H). Notably, qPCR analysis (S2 Table) revealed that the majority of particles (> 50%) had yet to release their genomes by 120 mpi (post-addition of virus). As vDNA is undetectable within native capsids (S2 Fig), these data suggest that the process of nuclear infection is still ongoing at 120 mpi. We note that under equivalent MOI conditions, ΔICP0^EdU^ infected cells had a reduced number of vDNA positive nuclei at each time point (Fig 1F–1H), a phenotype that likely reflects the efficiency of ICP0-null mutant EdU vDNA labelling in restrictive RPE cells (S1G–S1I Fig).

### PML-NBs entrap WT HSV-1 genomes upon nuclear entry

**Fig 2:**
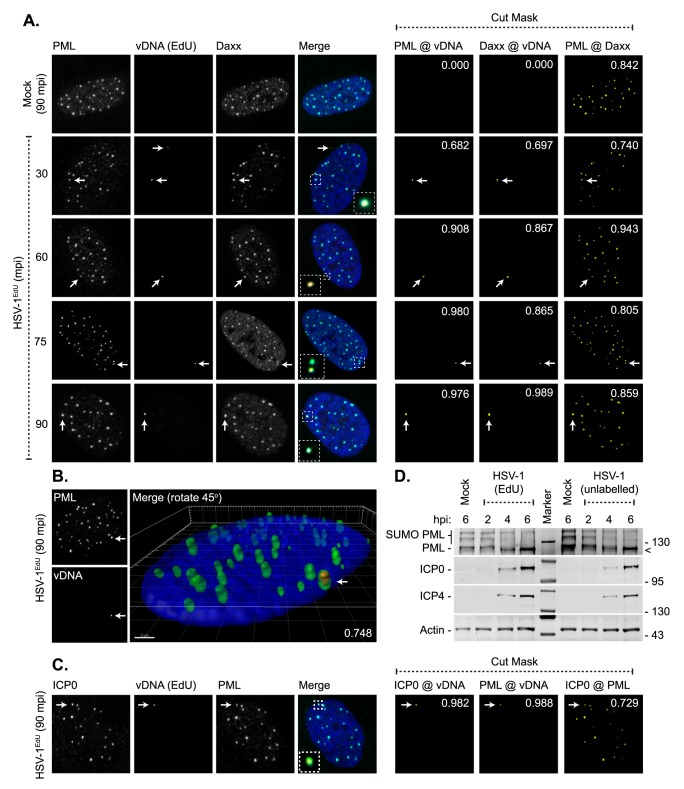
PML-NBs stably entrap vDNA upon nuclear entry. HFt cells were mock or HSV-1^EdU^ infected at an MOI of 3 PFU/cell. Cells were fixed and permeabilized at the indicated times (mpi; post-addition of virus). vDNA, or PML-NB host factors and ICP0, were detected by click chemistry and indirect immunofluorescence staining, respectively. (A) Localization of PML (green) and Daxx (cyan) to HSV-1 vDNA (red, white arrows) at 30–90 mpi (as indicated). Insets show magnified regions of interest (dashed boxes) highlighting PML-NB colocalization with vDNA. Cut mask (yellow) highlights regions of colocalization between PML, Daxx, and vDNA (as indicated). Weighted colocalization coefficients are shown. (B) 3D reconstruction of high-resolution confocal Z-series images showing PML (green) entrapment of vDNA (red, white arrow) at 90 mpi. Single channel maximum intensity projections (left-hand panels) and 3D rendered image (right-hand panel). Pearson coefficient for PML-vDNA colocalization is shown. Scale bar 2 μm. (C) Nuclear localization of ICP0 (green) to vDNA (red, white arrow) and PML (cyan) in HSV-1^EdU^ infected cells at 90 mpi. Cut mask (yellow) highlights regions of colocalization between ICP0, PML, and vDNA (as indicated). Weighted colocalization coefficients are shown. Nuclei stained with DAPI (blue). (D) EdU labelling of viral genomes does not affect the initiation of infection. HFt cells were mock or HSV-1 infected with unlabelled (HSV-1) or EdU labelled (HSV-1^EdU^) HSV-1 at an MOI of 3 PFU/cell. Whole cell lysates were collected at the indicated times (hours post-infection; hpi) for western blot analysis to monitor the rate of PML degradation and the accumulation of the viral immediate early (IE) gene products (ICP0, ICP4). Actin is shown as a loading control. Molecular mass markers are shown, < denotes the detection of a non-specific background band.

With the ability to detect input vDNA within the nuclei of infected cells as early as 30 mpi (post-addition of virus), we next assessed the utility of this approach to investigate the recruitment of intrinsic immune factors to infecting WT HSV-1^EdU^ genomes over a short time-course of infection (30 to 90 mpi; Fig 2). Using a combination of click chemistry to detect vDNA and immuno-labelling to detect PML-NB intrinsic host factors, PML (the main scaffolding protein of PML-NBs; [48]) and Daxx (a core constituent protein of PML-NBs; [48]) were observed to stably colocalize with vDNA over the time course of infection (30–90 mpi; Fig 2A). High-resolution Z-series imaging revealed input vDNA to be encased in PML following nuclear infection (Fig 2B). At 90 mpi, ICP0 could be observed to colocalize with PML-NBs prior to PML degradation and PML-NB disruption (Fig 2C and 2D; [22, 25–27]). ICP0 localization at multiple PML-NBs demonstrates that vDNA entrapment within any single PML-NB is not sufficient to target ICP0 to that specific body (Fig 2C). Western blotting of infected cell lysates demonstrated that EdU labelling of vDNA was not detrimental to the initiation of IE gene expression (ICP0, ICP4) or the degradation of PML (Fig 2D). Collectively, these data that demonstrate infecting WT HSV-1 genomes are rapidly encased by PML-NB intrinsic host factors from the outset of nuclear infection prior to the onset of lytic replication. Our data contrast with previous recruitment studies, which have reported PML-NB constituent proteins to localize to sites in close proximity to infecting viral genomes [8–10, 40]. However, we note that these studies have typically relied on higher MOI conditions (≥ 10 PFU/cell; [10]), the use of vDNA binding proteins to enable genome detection by proxy, or time points post-infection where vDNA replication proteins are readily detectable. We conclude that PML-NB host factors rapidly entrap viral genomes shortly after nuclear entry prior to the robust onset of viral gene expression (Fig 2D).

**Fig 3:**
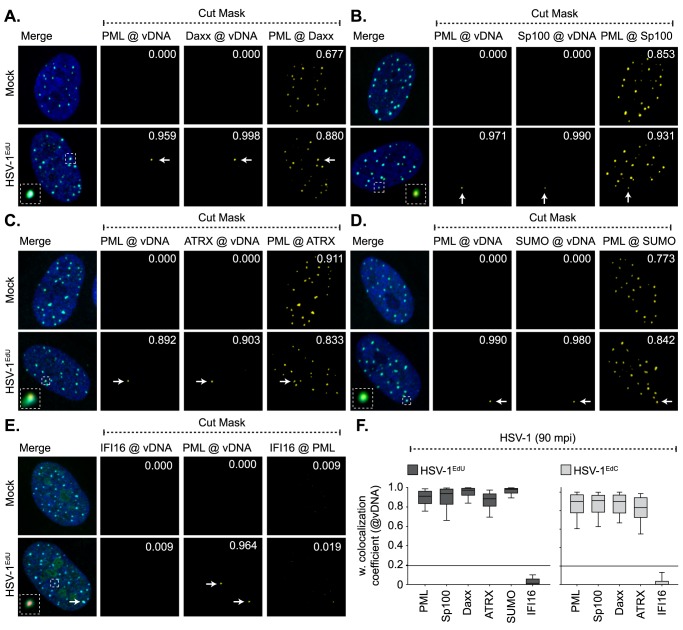
Core PML-NB proteins, but not IFI16, associate with infecting HSV-1 genomes. HFt cells were mock or infected with either HSV-1^EdU^ or HSV-1^EdC^ at an MOI of 3 PFU/cell. Cells were fixed and permeabilized at the indicated times (mpi; post-addition of virus). vDNA and PML-NB host factors were detected by click chemistry and indirect immunofluorescence staining, respectively. (A-E) Localization of PML (green), and either Daxx, Sp100, ATRX, SUMO2/3 (SUMO), or IFI16 (cyan; as indicated) to infecting HSV-1^EdU^ vDNA (red, white arrows). Insets show magnified regions of interest (dashed boxes) highlighting host protein localization with vDNA. Cut mask (yellow) highlights regions of colocalization between host proteins and vDNA (as indicated). Weighted colocalization coefficients are shown. Individual channel images shown in S3 Fig. Nuclei were stained with DAPI (blue). (F) Quantitation of host protein recruitment to infecting viral genomes labelled with EdU (as shown in A-E) or EdC. Boxes: 25^th^ to 75^th^ percentile range; black line: median weighted (w.) colocalization coefficient; whiskers: 5^th^ to 95^th^ percentile range. Solid line indicates coincident threshold level (weighted colocalization coefficients < 0.2). n ≥ 50 vDNA foci per sample population from a minimum of three independent infections.

Asynchronous plaque-edge recruitment studies have shown that PML-NB host factors are independently recruited to infecting viral genomes [49, 50] in an IFI16-dependent manner [11, 12, 14], where *de novo* PML-NB like foci are reformed [10]. We therefore assessed the composition of vDNA containing PML-NBs, as well as the localization of the PRR IFI16, to infecting WT HSV-1^EdU^ or HSV-1^EdC^ genomes (Fig 3, S3 Fig). High colocalization frequencies (weighted colocalization coefficients > 0.7) were observed for all PML-NB component proteins examined (PML, Daxx, Sp100, ATRX, and SUMO2/3) to infecting viral genomes irrespective of genome label, with equivalent paired colocalization frequencies observed for resident PML-NB proteins in mock-infected cells (Fig 3A–3D and 3F, S3 Fig). These data demonstrate that PML-NBs that contained vDNA were indistinguishable in composition from other PML-NBs within the same infected cell or in mock-infected cells at 90 mpi. Surprisingly, the colocalization frequency between IFI16 and input viral genomes was below coincident threshold levels (weighted colocalization coefficients < 0.2; solid line), demonstrating that IFI16 does not stably localize with viral genomes entrapped within PML-NBs at 90 mpi (Fig 3E and 3F, S3 Fig). These data again contrast with published asynchronous plaque-edge recruitment studies that have used vDNA binding proteins for genome detection, which have shown IFI16 and PML to both localize to infecting HSV-1 ICP0-null mutant genomes [11, 13, 14]. As ICP0 has been reported to promote the degradation of IFI16 [11, 28–31], we examined the recruitment of IFI16 to ΔICP0^EdU^ genomes to investigate the potential effect of ICP0 expression on IFI16 localization to vDNA. No stable localization of IFI16 could be observed to infecting ΔICP0^EdU^ genomes (S4 Fig), demonstrating that a lack of stable IFI16 recruitment to viral genomes was not due to low levels of ICP0 expression at 90 mpi (Fig 2C). We note that IFI16 localization to viral genomes has been reported to be highly dynamic [12, 14], which could potentially be inhibited by PML-NB entrapment of vDNA. We therefore investigated the influence of MOI (1, 10, and 50 PFU/cell) and time (15 or 30 mpi) on the recruitment of IFI16 and PML to nuclear infecting HSV-1^EdU^ genomes (S5 Fig). While vDNA-IFI16 colocalization could be observed under very high MOI conditions (50 PFU/cell; S5A Fig), quantitation (n ≥ 250 genomes) revealed the frequency of these colocalization events was not significantly altered by MOI or time (S5B–S5E Fig). In contrast, PML colocalization with vDNA was significantly reduced in a MOI dependent manner (1–50 PFU/cell), indicative of PML-NB saturation by viral genomes or disruption by ICP0 under these high MOI conditions. PML recruitment to vDNA also occurred in a time dependent manner, with a significant increase in colocalization frequency between 15 and 30 mpi (MOI of 10 PFU/cell). As bio-orthogonal nucleic acid labelling is incompatible with live-cell kinetic studies, we conclude that IFI16 does not form a stable association with input vDNA following genome entry into the nucleus.

### IFI16 recruitment to input viral genomes is not enhanced in the absence of PML

**Fig 4:**
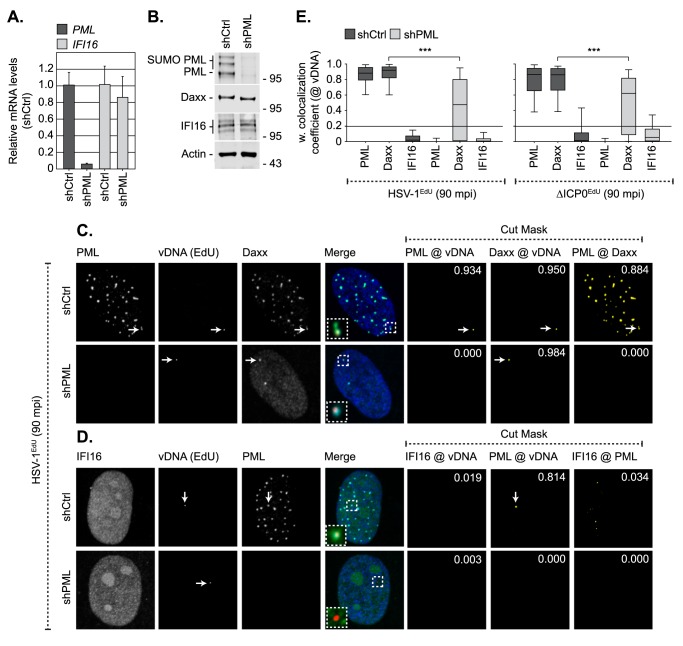
Depletion of PML does not enhance the recruitment of IFI16 to infecting viral genomes. HFt cells were stably transduced with lentiviruses expressing PML-targeting (shPML) or non-targeting control (shCtrl) shRNAs. (A) qRT-PCR quantitation of *PML* or *IFI16* mRNA levels in HFt shCtrl and shPML cells. Mean (RQ) and standard deviation (RQmin/max) shown and expressed relative to HFt shCtrl cells (1). (B) Western blot analysis of the expression levels of PML, Daxx and IFI16 in whole cell lysates derived from HFt shCtrl and shPML cells. Actin is shown as a loading control. Molecular mass markers are shown. (C, D) Localization of PML (green), and either Daxx or IFI16 (cyan; as indicated), to infecting HSV-1^EdU^ vDNA (red, white arrows) in HFt shCtrl and shPML cells at 90 mpi (post-addition of virus). Cells were infected with HSV-1^EdU^ or ΔICP0^EdU^ at an MOI of 3 PFU/cell. Insets show magnified regions of interest (dashed boxes) highlighting host protein localization with vDNA. Cut mask (yellow) highlights regions of colocalization between PML, IFI16, Daxx, and vDNA (as indicated). Weighted colocalization coefficients are shown. Nuclei were stained with DAPI (blue). Images for ΔICP0^EdU^ infected cells are shown in S6 Fig. (E) Quantitation of host protein recruitment to infecting viral genomes (as shown in C, D, S6). Boxes: 25^th^ to 75^th^ percentile range; black line: median weighted (w.) colocalization coefficient; whiskers: 5^th^ to 95^th^ percentile range. Solid line indicates coincident threshold level (weighted colocalization coefficients < 0.2). n ≥ 50 vDNA foci per sample population from a minimum of four independent infections. *** *P* < 0.001, Mann-Whitney *U*-test.

To test if PML-NBs competitively exclude IFI16 from binding vDNA following nuclear entry, we investigated the recruitment of IFI16 and Daxx (as a positive control; [49]) to input HSV-1^EdU^ and ΔICP0^EdU^ genomes in cells depleted of PML (Fig 4, S6 Fig). HFt cells were stably transduced with lentiviral vectors expressing non-targeting control or PML-targeting short hairpin RNAs (shCtrl and shPML, respectively; [49]). qRT-PCR and western blotting confirmed PML depletion without influencing Daxx or IFI16 expression (Fig 4A and 4B). HSV-1^EdU^ or ΔICP0^EdU^ infection of shCtrl cells recapitulated observations made in parental HFt cells (Fig 3, S3 Fig, S4 Fig), demonstrating that lentiviral transduction, shRNA expression, or puromycin selection did not affect PML-NB entrapment of vDNA or alter IFI16 localization (Fig 4C–4E). PML depletion did not increase the frequency of IFI16 colocalization with vDNA during either HSV-1^EdU^ or ΔICP0^EdU^ infection (Fig 4D and 4E, S6 Fig), demonstrating that PML-NBs do not competitively exclude IFI16 from binding vDNA. In contrast, while Daxx colocalized with vDNA in a subset of PML depleted cells (Fig 4C, S6A Fig), quantitation (n ≥ 100 genomes) revealed that the frequency of this colocalization was significantly reduced compared to control cells irrespective of ICP0 expression (Fig 4E). These data contrast with asynchronous plaque-edge recruitment studies, where Daxx recruitment to infecting viral genomes occurs in a PML independent manner under high MOI conditions [49]. We conclude that under infection conditions that do not saturate or disrupt PML-NBs by 90 mpi (MOI < 10 PFU/cell; S5 Fig), Daxx colocalization with vDNA is stabilized by PML at PML-NBs where Daxx is a resident protein (Figs 2–4, S3 Fig; [48]).

**Fig 5:**
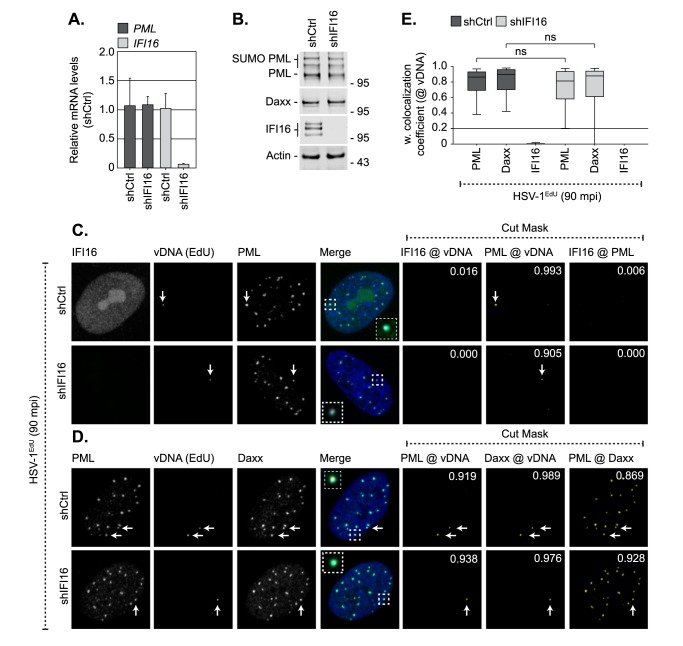
IFI16 does not influence the recruitment of PML or Daxx to infecting viral genomes. HFt cells were stably transduced with lentiviruses expressing IFI16-targeting (shIFI16) or non-targeting control (shCtrl) shRNAs. (A) qRT-PCR quantitation of *IFI16* or *PML* mRNA levels in HFt shCtrl and shIFI16 cells. Mean (RQ) and standard deviation (RQmin/max) shown and expressed relative to HFt shCtrl cells (1). (B) Western blot analysis of the expression levels of IFI16, PML, and Daxx in whole cell lysates from HFt shCtrl and shIFI16 cells. Actin is shown as a loading control. Molecular mass markers are shown. (C, D) Localization of PML (green), and either IFI16 or Daxx (cyan; as indicated) to infecting HSV-1^EdU^ vDNA (red, white arrows) in HFt shCtrl and shIFI16 cells at 90 mpi (post-addition of virus). Cells were infected with HSV-1^EdU^ at an MOI of 3 PFU/cell. Insets show magnified regions of interest (dashed boxes) highlighting host protein localization with vDNA. Cut mask (yellow) highlights regions of colocalization between IFI16, PML, Daxx, and vDNA (as indicated). Weighted colocalization coefficients are shown. Nuclei were stained with DAPI (blue). (E) Quantitation of host protein recruitment to infecting viral genomes (as shown in C, D). Boxes: 25^th^ to 75^th^ percentile range; black line: median weighted (w.) colocalization coefficient; whiskers: 5^th^ to 95^th^ percentile range. Solid line indicates coincident threshold level (weighted colocalization coefficients < 0.2). n ≥ 50 vDNA foci per sample population from a minimum of four independent infections. ns (not significant), Mann-Whitney *U*-test.

Live cell microscopy studies and asynchronous plaque-edge recruitment assays have reported that IFI16 is required for PML and Daxx recruitment to infecting viral genomes [11, 12, 14], although no direct evidence of IFI16 colocalization with vDNA or deposition within PML-NBs was reported to support this hypothesis. We therefore investigated if IFI16 played a role in PML-NB entrapment of vDNA (Fig 5). HFt cells were stably transduced with lentiviral vectors expressing non-targeting control or IFI16-targeting short hairpin RNAs (shCtrl and shIFI16, respectively; [11]). qRT-PCR and western blotting confirmed IFI16 depletion without influencing PML or Daxx expression (Fig 5A and 5B). HSV-1^EdU^ infection of shCtrl or shIFI16 cells demonstrated that both PML and Daxx strongly colocalized with vDNA independently of IFI16 (Fig 5C–5E). We conclude that IFI16 does not play an essential role in the entrapment of vDNA within PML-NBs. As we failed to observe any significant recruitment of IFI16 to infecting HSV-1 genomes under a range of infection conditions, our data suggest that the stable recruitment of PML and IFI16 to infecting viral genomes occurs with temporally distinct kinetics.

### vDNA entry into the nucleus is not sufficient to induce ISG expression

**Fig 6:**
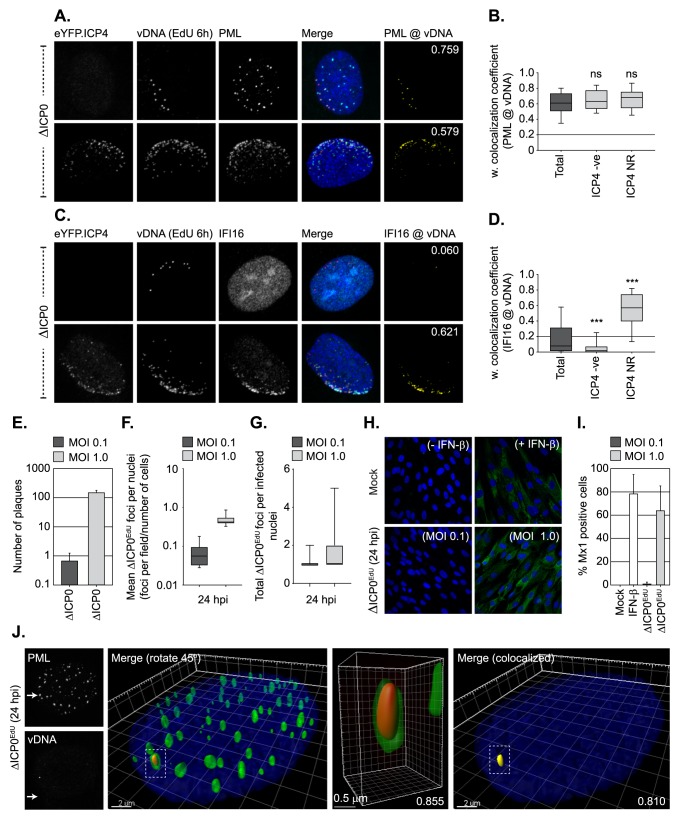
Nuclear entry of vDNA is not sufficient to induce robust Mx1 ISG expression. (A-D) Recruitment of PML, IFI16, and eYFP.ICP4 to ΔICP0 infecting viral genomes in an asynchronous plaque-edge recruitment assay (as described in [10]). HFt cells were infected with ΔICP0 expressing eYFP.ICP4 under conditions (MOI 2 PFU/cell) that enable plaque-formation to occur. Cells were pulse labelled at 24 hpi with 1 μM EdU for 6h to label vDNA. (A, C) Localization of eYFP.ICP4 (green), PML or IFI16 (cyan; as indicated) to vDNA (red) in newly infected cells on the periphery of a developing plaque-edge. Cut mask (yellow) highlights regions of colocalization between either PML or IFI16 and vDNA (as indicated). Weighted (w.) colocalization coefficients shown. (B, D) Quantitation of PML and IFI16 recruitment to infecting vDNA in the presence or absence of eYFP.ICP4 at the nuclear rim (ICP4 NR and ICP4 -ve, respectively; as shown in A and C). Boxes: 25^th^ to 75^th^ percentile range; black line: median weighted (w.) colocalization coefficient; whiskers: 5^th^ to 95^th^ percentile range. Solid line indicates coincident threshold level (weighted colocalization coefficients < 0.2). n = 100 plaque-edge cells +ve for vDNA per PML or IFI16 sample population from 4 independent infections. *** *P* < 0.001, ns (not significant), Mann-Whitney *U*-test. (E-I) Mx1 expression is only induced under infection conditions that permit ΔICP0 plaque formation. (E) Number of plaques detected at 24 hpi following ΔICP0 infection of HFt cells at an input MOI of 0.1 or 1 PFU/cell (as indicated). n = 3, means and standard deviations shown. (F) Quantitation of ΔICP0^EdU^ nuclear genomes in HFt cells (as infected in E). Boxes: 25^th^ to 75^th^ percentile range; black line: median; whiskers: 5^th^ to 95^th^ percentile range. n ≥ 250 cells derived from 3 independent experiments per condition. (G) Frequency of ΔICP0^EdU^ genomes detected within infected cell nuclei (as described in E/F). Boxes: 25^th^ to 75^th^ percentile range; black line: median; whiskers: minimum and maximum range of sample. (H) HFt cells were mock treated, IFN-β stimulated (100 IU/ml), or infected with ΔICP0 at a MOI of 0.1 or 1 PFU/cell. Samples were fixed at 24 post-treatment and analysed by confocal microscopy for Mx1 (green) ISG expression. (I) Quantitation of Mx1 positive cells (as shown in H). n ≥ 250 cells derived from 3 independent experiments per condition. (J) PML-NB entrapment of vDNA is maintained under low MOI conditions (0.1 PFU/cell) that restrict ΔICP0^EdU^ replication and plaque formation at 24 hpi. 3D reconstruction of high-resolution Z-series confocal images showing PML entrapment of ΔICP0^EdU^ vDNA. Single channel maximum intensity projection images (left), 3D rendered images of the whole nucleus showing PML (green) and vDNA (red), with a single vDNA focus entrapped by PML (dashed box; right). Scale bar 2 μm. Enlargement of PML entrapped vDNA (centre-right). Scale bar 0.5 μm. Cut mask (yellow) highlights colocalization between PML and vDNA (far-right). Pearson coefficient for PML-vDNA colocalization shown. Nuclei were stained with DAPI (blue).

As asynchronous plaque-edge recruitment assays have played a key role in defining the recruitment of IFI16 to infecting HSV-1 genomes [11, 13, 14], we conducted analogous assays to assess the temporal recruitment of PML and IFI16 to both vDNA and ICP4, an IE vDNA binding protein commonly utilized as a proxy for vDNA in genome recruitment studies [9–11, 14, 40]. Viral DNA labelling was achieved by pulse labelling HSV-1 ICP0-null mutant infected cell monolayers at 24 hours post-infection (hpi) with 1 μM EdU for 6 h. Under these conditions, DNA replication compartments within the body of a developing plaque were clearly detected (S7 Fig). Cells on the periphery of the plaque-edge were readily observed to contain EdU positive vDNA foci asymmetrically distributed around the nuclear rim prior to ICP4 detection (Figs 6A, 6C top panels, S7), indicative of input EdU labelled viral genomes that have yet to initiate a productive gene expression programme. The recruitment of PML to infecting viral genomes occurred independently of ICP4 expression, with many genome foci observed to localise in close proximity to PML foci (Fig 6A and 6B). In contrast, IFI16 recruitment only occurred in cells that expressed ICP4 localized to vDNA at the nuclear rim (ICP4 NR; Fig 6C and 6D, S7 Fig). These data support previous asynchronous recruitment studies that have used ICP4 as a proxy for genome detection [11, 14] and demonstrate that PML and IFI16 are recruited to infecting viral genomes with temporally distinct kinetics, which in the case of IFI16 correlates with the expression and localization of viral gene products with vDNA (Fig 6D). Importantly, these data suggest that vDNA entry into the nucleus alone may not be sufficient to stimulate the induction of an IFI16-dependent innate immune response, but instead require the expression of specific viral gene products or the initiation of vDNA replication.

To test this hypothesis, we examined the induction of Mx1, a well-characterized ISG product [51], during HSV-1 ICP0-null mutant infection by confocal microscopy. HFt cells were mock treated, stimulated with IFN-β (as a positive control), or infected with ΔICP0^EdU^ at input levels which either restrict or permit the initiation of HSV-1 ICP0-null mutant replication and plaque formation at 24 hpi (MOI 0.1 and 1.0 PFU/cell, respectively; Fig 6E). vDNA was readily detectible within the nuclei of infected cells under restrictive MOI conditions (0.1 PFU/cell) with a frequency close its expected input genome ratio (Fig 6F) and copy number (1–2 genomes/infected cell; Fig 6G, S2 Table). Notably, the number of genomes per infected cell nuclei under permissive conditions (MOI 1 PFU/cell) was lower than expected based on our input qPCR analysis (~ 25 genomes/cell; Fig 6G, S2 Table). These data indicate that under MOI conditions that begin to saturate intrinsic host defences (~ 10–20 genome copies/nuclei), ΔICP0^EdU^ genome detection is lost following the onset of vDNA replication by 24 hpi.

As expected, IFN-β stimulation efficiently induced Mx1 expression 24 h post-treatment (Fig 6H and 6I). In contrast, infection with ICP0-null mutant HSV-1 only stimulated Mx1 expression at genome input levels sufficient to stimulate the onset of viral replication and plaque formation (MOI 1.0 PFU/cell; Fig 6E, 6H and 6I). High-resolution Z-series imaging revealed that viral genomes remained stably entrapped within PML-NBs under restrictive MOI conditions (0.1 PFU/cell) at 24 hpi (Fig 6J). These data demonstrate that under infection conditions that restrict the initiation of ICP0-null mutant HSV-1 replication, viral genome entry into the nucleus alone is not sufficient to stimulate the induction Mx1 ISG expression.

### vDNA replication is required for the induction of innate immunity

**Fig 7:**
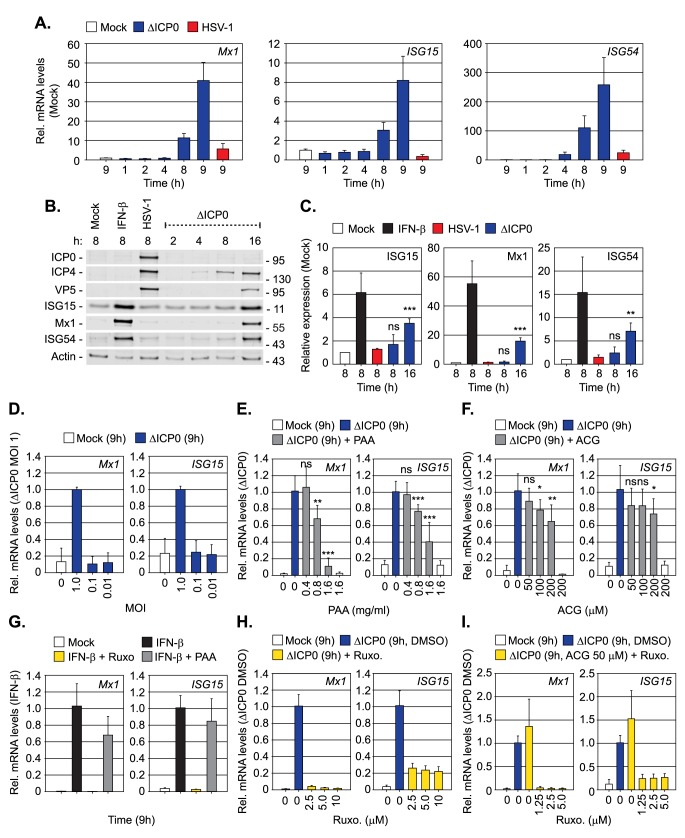
Inhibition of vDNA replication impairs the induction of innate immunity during HSV-1 infection. HFt cells were mock, WT, or ΔICP0 HSV-1 infected at an MOI of 1 PFU/cell (unless stated otherwise). Samples were collected at the indicated times (hours; h) post-treatment or infection. (A) Relative mRNA levels of *Mx1*, *ISG15*, and *ISG54* during WT or ΔICP0 HSV-1 infection. n = 2, means (RQ) and standard deviations (RQmin/max) are shown and expressed relative to mock (1). (B) Western blot analysis of the expression levels of ISGs (ISG15, Mx1, ISG54), viral proteins (ICP0, ICP4, VP5), and actin (as a loading control), from whole cell lysates of mock, IFN-β stimulated (100 IU/ml), WT or ΔICP0 HSV-1 infected HFt cells at the indicated times. Molecular mass markers are shown. (C) Quantitation of ISG expression levels (as shown in B). n = 3, means and standard deviations shown expressed relative to mock (1). ** *P* < 0.01, *** *P* < 0.001, ns (not significant); two-tailed t-test. (D) Relative mRNA levels of *Mx1* and *ISG15* in mock or ΔICP0 infected HFt cells (MOI of 0.01 to 1 PFU/cell, as indicated). n = 3, means (RQ) and standard deviations (RQmin/max) are shown and expressed relative to ΔICP0 MOI 1 (1). (E, F) Relative mRNA levels of *Mx1* and *ISG15* in mock or ΔICP0 infected HFt cells in the presence of phosphonoacetic acid (PAA) or acycloguanosine (ACG) at the indicated concentrations. n = 3, means (RQ) and standard deviations (RQmin/max) are shown and expressed relative to ΔICP0 (1). * *P* < 0.05, ** *P* < 0.01, *** *P* < 0.001, ns (not significant); two-tailed t-test. (G) Relative mRNA levels of *Mx1* and *ISG15* in mock or IFN-β stimulated (100 IU/ml) HFt cells co-incubated in the presence Ruxolitinib (5 μM; Ruxo) or PAA (1.6 mg/ml), as indicated. n = 2, means (RQ) and standard deviations (RQmin/max) are shown and expressed relative to IFN-β treatment (1). (H, I) Relative *Mx1* and *ISG15* mRNA levels in mock or ΔICP0 infected HFt cells treated with Ruxo (2.5–10 μM, as indicated) or Ruxo (5 μM) and ACG (50 μM), as indicated. n = 2, means (RQ) and standard deviations (RQmin/max) are shown and expressed relative to ΔICP0 infection (1).

As PML-NB entrapped HSV-1 ICP0-null mutant genomes failed to stimulate the induction of Mx1 expression, we next examined the kinetics of ISG induction during HSV-1 infection under MOI conditions that enabled the onset of viral replication (MOI 1 PFU/cell; Fig 7). As expected, HSV-1 ICP0-null mutant infection efficiently induced the transcription (by 8–9 hpi) and expression (by 16 hpi) of three independent ISG products (*Mx1*, *ISG15*, and *ISG54*), a host response that was significantly impaired during WT HSV-1 infection (Fig 7A–7C). Importantly, the induction of ISGs only occurred under infection conditions that enabled the onset of ICP0-null mutant HSV-1 replication and plaque-formation (≥ 1.0 PFU/cell; Figs 6E and 7D). Consistent with our microscopy observations (Fig 6H and 6I), these data demonstrate that saturation of intrinsic host defences is required for the robust induction of ISGs during HSV-1 ICP0-null mutant infection. As IFI16 binds to a range of DNA structures that may be produced during vDNA replication [52], we next investigated the role of vDNA replication in the induction of ISGs. ISG transcript levels were monitored in the presence of the vDNA replication inhibitors PAA (phosphonoacetic acid) and ACG (acycloguanosine), two well-characterized herpesvirus DNA replication inhibitors [53–57]. PAA efficiently inhibited the induction of both *Mx1* and *ISG15* transcript levels in a dose-dependent manner (Fig 7E), while ACG treatment had only a modest inhibitory effect at concentrations sufficient to restrict HSV-1 plaque formation (≥ 50 μM; Fig 7F, S1 Table). Inhibition of ISG induction by PAA was virus-specific, as ISG transcript levels were readily induced by IFN-β stimulation in the presence of PAA (Fig 7G). By way of contrast, JAK inhibition by Ruxolitinib (Ruxo) effectively blocked ISG induction following IFN-β stimulation (Fig 7G; [58, 59]), consistent with a key role for JAK in IFN-mediated innate immune signalling [60]. JAK inhibition also effectively blocked ISG induction during HSV-1 ICP0-null mutant infection (Fig 7H). Importantly, this inhibition occurred in the presence 50 μM ACG (Fig 7I), demonstrating that JAK activity is specifically required to induce ISG expression during the initiating cycle(s) of HSV-1 ICP0-null mutant vDNA replication by 9 hpi. Together with our microscopy observations (Fig 6), these data demonstrate that the onset of vDNA replication is required for the robust induction of ISG expression during HSV-1 ICP0-null mutant infection.

### Dual roles for both PML and IFI16 in the regulation of intrinsic and innate immunity to HSV-1 infection

**Fig 8:**
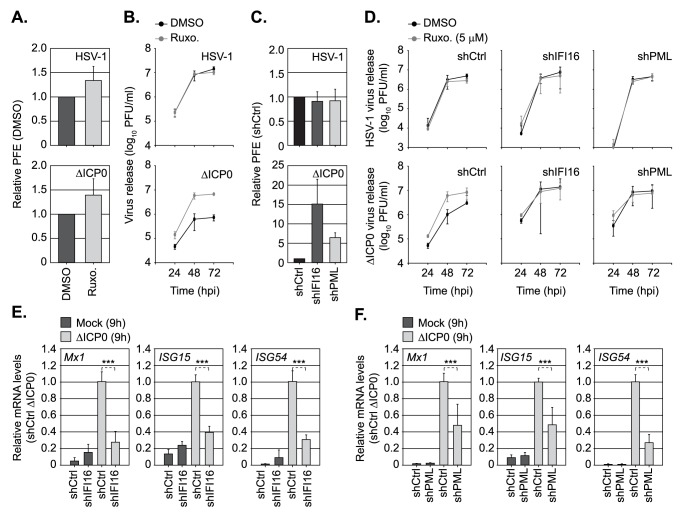
Dual roles for PML and IFI16 in the regulation of intrinsic and innate immunity to HSV-1 infection. (A) HFt cells were DMSO or Ruxolitinib (5μM; Ruxo) treated and infected with serial dilutions of WT or ΔICP0 HSV-1 for 24 h prior to immuno-staining for plaque formation. Plaque counts expressed relative to control cell monolayers (# of plaques treated / # of plaques DMSO control) at equivalent serial dilutions of virus and presented as relative plaque formation efficiency (PFE). n ≥ 3, means and standard deviations shown. (B) HFt cells were DMSO or Ruxo treated and infected with WT (MOI 0.001 PFU/cell) or ΔICP0 (MOI 1 PFU/cell) HSV-1. Cell released virus (CRV) was harvested at the indicated times (hpi) and CRV titres determined on U2OS cells. n = 3, means and standard deviations shown. (C) Stably transduced HFt cells expressing non-targeting control (shCtrl), or targeting IFI16 (shIFI16) or PML (shPML) shRNAs, were infected with WT or ΔICP0 HSV-1 (as in A). Plaque counts expressed relative to infected shCtrl cell monolayer plaque counts (1) and presented as relative PFE. n = 3, means and standard deviations shown. (D) shCtrl, shIFI16, or shPML HFt cells were treated with DMSO or Ruxo and infected with either WT or ΔICP0 HSV-1 (as in B). CRV was collected at the indicated times (hpi) and titres determined on U2OS cells. n = 3, means and standard deviations shown. (E, F) Relative *Mx1*, *ISG15*, and *ISG54* mRNA levels in HFt shCtrl, shIFI16, or shPML cells mock or ΔICP0 infected (MOI 1 PFU/cell) at 9 hpi. n = 3, means (RQ) and standard deviations (RQmin/max) shown and expressed relative to ΔICP0 infected shCtrl (1). *** *P* < 0.001; two-tailed t-test.

As the induction of innate immunity during HSV-1 ICP0-null mutant infection was inhibited by Ruxolitinib, we next examined the effect of JAK inhibition on WT and ICP0-null mutant HSV-1 replication (Fig 8). At a concentration sufficient to inhibit ISG induction (5 μM; Fig 7G–7I), Ruxolitinib treatment had no effect on the relative plaque formation efficiency (PFE) of either WT or ICP0-null mutant HSV-1 (Fig 8A). These data indicate that innate immune signalling and the induction of ISGs does not directly contribute to the cellular restriction and plaque-formation defect of an HSV-1 ICP0-null mutant observed in restrictive cell types (≥ 1000 fold; [33, 35, 61]). In contrast, virus yield assays demonstrated that Ruxolitinib treatment enhanced the levels of ICP0-null mutant, but not WT, HSV-1 propagation (Fig 8B). Thus, under infection conditions that saturate intrinsic host defences and enable the onset of HSV-1 ICP0-null mutant replication (MOI of ≥ 1 PFU/cell; Fig 6E), innate immune defences act to restrict virus propagation. By way of contrast, depletion of IFI16 or PML enhanced both the PFE and virus yield of an HSV-1 ICP0-null mutant (Fig 8C and 8D; [11, 49]). Importantly, no additional increase in virus yield was observed on Ruxolitinib treatment of IFI16 or PML depleted cells (Fig 8D), indicative of an impaired innate immune response in these cells during HSV-1 ICP0-null mutant infection. Correspondingly, qRT-PCR demonstrated that the induction of ISGs (*Mx1*, *ISG15*, *ISG54*) was significantly impaired in both IFI16 or PML depleted cells in response to HSV-1 ICP0-null mutant infection at 9 hpi (Fig 8E and 8F). These data identify a novel role for PML in the induction of ISGs and the regulation of innate immunity during HSV-1 infection. Collectively, our data demonstrate that PML plays dual roles in the temporal regulation of intrinsic and innate immune defences that are dependent on viral genome delivery to the nucleus and the onset of vDNA replication, respectively.

**Fig 9:**
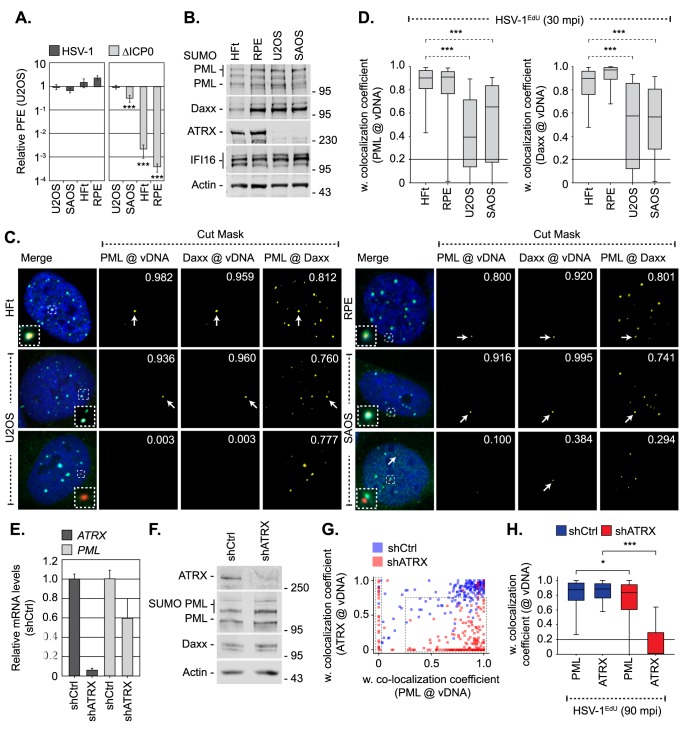
PML-NB entrapment of infecting HSV-1 vDNA occurs in a cell type and ATRX dependent manner. (A) U2OS, SAOS, RPE, and HFt cells were infected with serial dilutions of WT or ΔICP0 HSV-1 for 24 h. Plaque counts expressed relative to U2OS, a cell line permissive to ΔICP0 replication [34], (# of plaques / # of plaques U2OS control) at equivalent serial dilutions of virus and presented as relative plaque formation efficiency (PFE). n ≥ 3, means and standard deviations shown. (B) Western blot analysis of the relative expression levels of PML, Daxx, ATRX, and IFI16 in whole cell lysates derived from HFt, RPE, U2OS and SAOS cells. Actin is shown as a loading control. Molecular mass markers are indicated. (C) Localization of PML (green) and Daxx (cyan) to HSV-1^EdU^ vDNA (red, white arrows) in restrictive (HFt, RPE) and permissive (U2OS, SAOS) cell types at 30 mpi (post-addition of virus; MOI of 3 PFU/cell). Insets show magnified regions of interest (dashed boxes) highlighting host protein localization with vDNA. Cut mask (yellow) highlights regions of colocalization between PML, Daxx, and vDNA (as indicated). Weighted (w.) colocalization coefficients shown. (D) Quantitation of PML and Daxx recruitment to infecting vDNA in restrictive (HFt, RPE) and permissive (U2OS, SAOS) cell lines (as shown in C). Boxes: 25^th^ to 75^th^ percentile range; black line: median weighted (w.) colocalization coefficient; whiskers: 5^th^ to 95^th^ percentile range. Solid line indicates coincident threshold level (weighted colocalization coefficients < 0.2). n ≥ 50 vDNA foci per sample population from 4 independent infections. (E-H) ATRX is required for efficient PML-NB entrapment of vDNA. HFt cells were stably transduced with lentiviruses expressing ATRX-targeting (shATRX) or non-targeting control (shCtrl) shRNAs. (E) qRT-PCR quantitation of *ATRX* or *PML* mRNA levels in HFt shCtrl and shATRX cells. Mean (RQ) and standard deviation (RQmin/max) shown and expressed relative to HFt shCtrl cells (1). (F) Western blot analysis of the relative expression levels of ATRX, PML, and Daxx in whole cell lysates from HFt shCtrl and shATRX cells. Actin is shown as a loading control. Molecular mass markers are shown. (G) Scatter plot showing paired w. colocalization coefficients of ATRX and PML to individual nuclear infecting viral genomes in shCtrl (blue) and shATRX (red) cells infected with HSV-1^EdU^ at an MOI of 3 PFU/cell at 90 mpi (post-addition of virus). n ≥ 200 genomes per sample population. Dotted boxes highlight genome populations identified to have altered distribution of colocalization frequency in comparison to infected shCtrl cells. (H) Quantitation of PML and ATRX recruitment to infecting viral genomes (as shown in G). Boxes: 25^th^ to 75^th^ percentile range; black line: median weighted (w.) colocalization coefficient; whiskers: 5^th^ to 95^th^ percentile range. Solid line indicates coincident threshold level (weighted colocalization coefficients < 0.2). * *P* < 0.05, *** *P* < 0.001; Mann-Whitney *U*-test.

As intrinsic and innate immune defences to HSV-1 infection are known to be cell-type dependent [34, 35, 62], we next investigated if there was a correlation between cell line permissiveness to HSV-1 ICP0-null mutant replication and the entrapment of viral genomes by PML-NB host factors (Fig 9). Relative to permissive osteosarcoma cells (U2OS, SAOS), which do not require ICP0 to stimulate the onset of HSV-1 replication [34], RPE cells demonstrated equivalent levels of HSV-1 ICP0-null mutant restriction to HFt cells (≥ 1000-fold reduction in PFE, Fig 9A). Western blot analysis revealed that all these cell lines expressed similar levels of PML, Daxx, and IFI16 (Fig 9B). However, infection with HSV-1^EdU^ demonstrated a significant reduction in the colocalization frequency of PML and Daxx to infecting viral genomes between permissive (U2OS, SAOS) and restrictive (HFt, RPE) cell-types (Fig 9C and 9D). Importantly, in many instances neither PML nor Daxx was observed to localize with infecting viral genomes in permissive cell types (Fig 9C, bottom panels). Thus, we have identified a correlation between the stable entrapment of vDNA by PML-NBs and the requirement for ICP0 to stimulate the efficient onset of HSV-1 infection in restrictive (HFt, RPE), but not permissive (U2OS, SAOS), cell-types. As U2OS and SAOS cells do not express ATRX (Fig 9B; [63, 64]), a known core constituent protein of PML-NBs and an intrinsic antiviral regulator to HSV-1 infection [64–66], we investigated the requirement for ATRX to mediate PML-NB entrapment of vDNA. HFt cells were stably transduced with lentiviral vectors expressing non-targeting control or ATRX-targeting short hairpin RNAs (shCtrl and shATRX, respectively; [65]). qRT-PCR and western blotting confirmed ATRX depletion, which had a modest effect on PML mRNA transcript levels without influencing PML or Daxx expression levels (Fig 9E and 9F). HSV-1^EdU^ infection of shCtrl or shATRX cells demonstrated that depletion of ATRX led to a distinct population of viral genomes with a reduced colocalization frequency with PML (left-hand dotted box; Fig 9G). Notably, low levels of ATRX colocalization were still observed with vDNA in a significant proportion of ATRX depleted cells (right-hand dotted box; Fig 9G). Quantitation (n ≥ 200 genomes per condition) revealed that there was a significant difference in PML recruitment to vDNA in ATRX depleted cells (Fig 9H). We conclude that ATRX, either directly or indirectly, contributes to the entrapment of vDNA within PML-NBs following nuclear entry.

**Fig 10:**
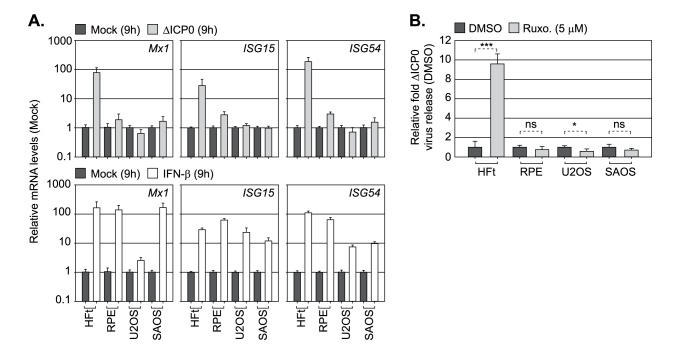
PML-NB entrapment of vDNA does not lead to the induction of innate immunity. (A) Relative *Mx1*, *ISG15*, and *ISG54* mRNA levels in cell lines restrictive (HFt, RPE) or permissive (U2OS, SAOS) to HSV-1 ICP0-null mutant (ΔICP0) infection. Cells were mock treated, IFN-β (100 IU/ml) stimulated, or infected with ΔICP0 at an MOI of 1 PFU/cell for 9 h. n = 3, means (RQ) and standard deviations (RQmin/max) are shown and expressed relative to mock for each cell line (1). (B) Restrictive (HFt, RPE) or permissive (U2OS, SAOS) cell lines were infected with ΔICP0 at an MOI of 1 PFU/cell and treated with either DMSO or Ruxolitinib (Ruxo, 5μM). CRV was harvested at 48 hpi and titres determined on U2OS cells. n = 3, means and standard deviations shown and expressed as relative fold-change to DMSO titres (1) for each cell line. * *P* < 0.05, *** *P* < 0.001, ns (not significant); two-tailed t-test.

Finally, we compared restrictive (HFt, RPE) and permissive (U2OS, SAOS; [62]) cell types to mount an innate immune response to HSV-1 ICP0-null mutant infection under infection conditions that permitted the onset of viral replication (MOI 1 PFU/cell). Surprisingly, qRT-PCR analysis demonstrated that only HFt cells induced ISG (*Mx1*, *ISG15*, *ISG54*) expression during HSV-1 ICP0-null mutant infection (Fig 10A, top panels; [62]). This was not due to a defect in IFN pathway signalling, as all four cell-types were responsive to exogenous IFN-β stimulation (Fig 10A, bottom panels). Correspondingly, only HFt cells showed enhanced levels of HSV-1 ICP0-null mutant propagation following JAK inhibition by Ruxolitinib (Fig 10B). We conclude that RPE cells, which are highly restrictive to HSV-1 ICP0-null mutant replication (Fig 9A), are defective in aspects relating to intracellular innate signalling in response to HSV-1 infection. These data support our microscopy observations (Fig 6) and inhibitor studies (Figs 7 and 8), and collectively demonstrate that intrinsic and innate host immune responses to HSV-1 infection are temporally distinct and functionally separable arms of host immunity.

In summary, we show that the temporal recruitment of host immune regulators to infecting viral genomes plays an important role in the sequential regulation of intrinsic and innate immunity during HSV-1 infection. We identify PML-NBs to entrap vDNA shortly after nuclear entry in an ATRX-dependent and IFI16-independent manner. We identify a novel role for PML in the induction of innate immunity in response to HSV-1 infection that correlates with the recruitment of IFI16 into vDNA complexes associated with ICP4 and the onset of vDNA replication. These intracellular host defences are counteracted by ICP0, which induces the degradation of PML from the outset of infection to release viral genomes entrapped within PML-NBs to stimulate the onset of HSV-1 lytic replication.

## Discussion

A key aspect in the regulation of intracellular host immunity during herpesvirus infection is the rapid recruitment of host immune factors to infecting viral genomes. This nuclear response to infection has been linked to viral genome silencing, as part of a pre-existing intrinsic immune defence, and the activation of innate immune signalling pathways (reviewed in [1, 4, 32]). However, the temporal recruitment of these host immune regulators to infecting viral genomes upon vDNA entry into the nucleus has remained poorly defined due to the technical challenges associated with low genome copy-number detection.

Microscopy studies have historically relied on the use of viral mutants, high MOI conditions, and vDNA binding proteins to investigate the recruitment of host immune regulators to infecting viral genomes. Whilst informative, such approaches can readily saturate intrinsic host defences that restrict the initiation of viral gene expression due to high input genome loads [35]. Consequently, the temporal sequence of events that influence the sequential regulation of intracellular host immunity upon vDNA entry into the nucleus has remained poorly defined, specifically during WT herpesvirus infections that express a full complement of immune antagonists. Here we quantitatively examine the recruitment of intrinsic and innate immune regulators to infecting WT and ICP0-null mutant HSV-1 genomes under a range of relatively low MOI conditions (≤ 3 PFU/cell) within the first 15–90 mpi (post-addition of virus). We show that PML, the principle scaffolding protein of PML-NBs [48], plays temporally distinct and functionally separable roles in the regulation of intrinsic and innate immune defences activated in response to HSV-1 infection through the entrapment of viral genomes (Figs 2 and 6) and the induction of ISG expression following the onset of vDNA replication (Fig 7), respectively. These observations reconcile many longstanding issues within the field as to the importance of PML and PML-NBs during primary herpesvirus infection and the requirement for ICP0 to stimulate the onset of HSV-1 lytic replication, as discussed below.

Immuno-FISH experiments conducted by Gerd Maul and colleagues over 20 years ago originally identified that infecting HSV-1 genomes localize in close proximity to PML-NBs under infection conditions that enabled detection of ICP8 [8], an essential component of the vDNA replication complex [67]. These pioneering observations have stimulated a field of research that has uncovered fundamental roles for many PML-NB associated proteins in the regulation of intracellular host immunity against a range of DNA and RNA viral pathogens (reviewed in [1, 20, 21, 68]). PML-NBs are highly dynamic nuclear sub-domains, with resident proteins (PML, Sp100, and Daxx) in constant exchange with the surrounding nucleoplasm [10]. Correspondingly, asynchronous plaque-edge recruitment assays (examples of which are shown in Fig 6, S7 Fig; [9]) have shown that many PML-NB component proteins re-localize to sites in close proximity to infecting HSV-1 genomes under high MOI conditions, where *de novo* PML-NB like foci are reformed [10]. These observations have set the paradigm for intrinsic immunity during herpesvirus infection, where pre-existing PML-NB host factors re-localize to infecting viral genomes to mediate the transcriptional repression of viral gene expression [19, 49, 50, 69]. However, recent live-cell microscopy studies have proposed an alternate mechanism, whereby vDNA becomes transiently associated with host factor(s) within the nucleoplasm (notably Daxx and IFI16; [12, 14]) prior to deposition at PML-NBs, although no evidence for Daxx or IFI16 colocalization with vDNA or deposition within PML-NBs was reported. Using click chemistry, we demonstrate for the first time that infecting WT and ICP0-null mutant HSV-1 genomes are rapidly entrapped within PML-NBs following nuclear entry (30–90 mpi; Figs 2 and 6J). While we cannot rule out a recruitment model of genome entrapment, specifically the re-localization of PML-NB host factors that are in immediate proximity to nuclear infecting viral genomes, our data support a deposition model as: (i) PML-NBs that contained vDNA were indistinguishable in composition from other PML-NBs within the same infected nucleus or mock-infected cells (Fig 3A–3D, S3 Fig); (ii) Depletion of PML reduced Daxx colocalization with vDNA, indicative of a transient association stabilized by PML at PML-NBs where Daxx is a resident protein (Fig 4E; [48, 49]); (iii) Depletion of ATRX, a binding partner of Daxx [70], reduced the frequency of PML colocalization with vDNA in a significant subset of infected cells (Fig 9E–9H). These observations support a deposition model of vDNA entrapment at pre-existing PML-NBs that contain a core complement of PML-NB host factors and implicate the Daxx/ATRX complex in this process, consistent with live-cell microscopy observations [12]. These observations are consistent with co-depletion experiments [19, 50, 69], which have shown PML-NB proteins to act cooperatively to restrict the initiation of HSV-1 ICP0-null mutant replication under low MOI conditions (≤ 1 PFU/cell, 6E; < 25 genome copies/cell, S2 Table). We therefore provide spatial context to these studies, as repressed viral genomes remain stably entrapped within PML-NBs at 24 hpi (Fig 6J), a host response that is impaired in cell types permissive to HSV-1 ICP0-null mutant replication which lack ATRX (U2OS, SAOS; Fig 9A–9D; [34, 63, 64]). Thus, we identify PML-NB entrapment of vDNA as a key intrinsic antiviral host defence to WT herpesvirus nuclear infection, a conclusion consistent with genome localization studies in HSV-1 latently infected cells by immuno-FISH [71–73]. We demonstrate that this intrinsic PML-NB host defence occurs in a range of restrictive cell types relevant to primary HSV-1 infection (Figs 2 and 9) and independently of the induction of ISG expression (Figs 6H–6J, 7D and 10A), demonstrating that this host response does not directly contribute to the sensing of viral nucleic acids that leads to the induction of innate immunity.

Our data highlights the importance of ICP0 to promote the onset of WT HSV-1 infection under low MOI conditions [33–35]. ICP0 is known to localize to PML-NBs from the outset of infection in a PML isoform and SUMO-dependent manner [17, 22, 74, 75], where it targets PML and other SUMO-modified component proteins for ubiquitination and proteasome-dependent degradation [17, 25–27, 74, 75]. As ICP0 does not preferentially localize to PML-NBs that contain vDNA (Fig 2C), our data indicate that these vDNA containing nuclear bodies are likely to be equivalent in their respective PML isoform composition and SUMO modification status at this extremely early stage of nuclear infection. Thus, cell-wide PML-NB disruption through ICP0 mediated degradation of PML ensures viral genome release and the dispersal of associated PML-NB host factors that repress the onset of viral gene expression [19, 49, 50, 66, 69]. This hypothesis is supported by our depletion experiments that show reduced Daxx localization with vDNA in PML depleted cells (Fig 4E), and accounts for why many PML-NB resident host factors known to restrict HSV-1 ICP0-null mutant replication (including Daxx, ATRX, and PIAS1) are not directly targeted for degradation by ICP0 [19, 66]. Thus, the correct complement of host factors within pre-existing PML-NBs is likely to play an important role in mediating the cellular restriction of viral gene expression from the outset of nuclear infection. These observations account for why many herpesviruses encode IE gene products that disrupt the structural organization of PML-NBs (reviewed in [1, 68]), and the respective abilities of these proteins to complement the replication and plaque-forming defect of an HSV-1 ICP0-null mutant in restrictive cell types [76–79].

Our observation that repressed HSV-1 ICP0-null mutant genomes remain stably entrapped within PML-NBs without inducing ISG expression (Figs 6E–6J and 7D) has significant implications with respect to host PRR sensing of vDNA and the regulation of innate immunity during herpesvirus infection. The sensing of foreign DNA and the activation of innate immune signalling pathways can occur through multiple pathways and PRRs, including TLR9, RIG-I, MAVS, AIM2, DNA-PK, cGAS, and IFI16 (reviewed in [4–6]). Of these, IFI16 has received significant attention due to its role as a vDNA PRR in the induction of ISG expression and type-I IFN production during herpesvirus infection [28, 29, 80–84]. Correspondingly, microscopy assays have shown IFI16 to be recruited to infecting HSV-1 genomes in a pyrin domain-dependent manner in association with PML-NB host factors [11, 12, 14]. This host response was initially reported to be antagonized by the ubiquitin ligase activity of ICP0 [28, 29], although subsequent studies have shown other viral and cellular factors are likely to be involved [11, 30, 31]. IFI16 recruitment studies have relied on the use of vDNA binding proteins to enable viral genome detection by proxy, or on extrapolation of altered patterns in IFI16 nuclear localization to infer IFI16-vDNA association in restrictive cell types. Thus, the temporal kinetics of IFI16 recruitment to vDNA and its subsequent association with PML-NB host factors has remained poorly defined, specifically under MOI conditions relevant to WT herpesvirus infections. In contrast to PML-NB host factors, we failed to observe any significant frequency of stable colocalization between IFI16 and vDNA up to 90 mpi (post-addition of virus; Figs 3 and 5, S5 Fig), even in the absence of PML or ICP0 (Fig 4E). While we cannot exclude the possibility of highly transient IFI16 interactions with vDNA (S5A Fig; [12, 14]), our data indicates that IFI16 does not play an essential role in the entrapment of viral genomes by PML-NBs (Fig 5; [11, 14]). Importantly, under infection conditions that do not saturate or antagonize intrinsic PML-NB host defences (HSV-1 ICP0-null mutant MOI < 1 PFU/cell, Fig 6E; < 25 genome copies/cell, S2 Table), we demonstrate vDNA entry into the nucleus alone is not sufficient to stimulate a robust innate immune response (Figs 6E–6J and 7D). Induction of innate immunity only occurred under MOI conditions sufficient to saturate intrinsic host defences leading to the onset of HSV-1 ICP0-null mutant replication and plaque-formation (MOI ≥ 1 PFU/cell; Figs 6E and 7D). Under such conditions, we identified a clear kinetic difference in the stable recruitment of PML and IFI16 to infecting viral genomes, which in the case of IFI16 correlated with the recruitment of ICP4 (the major IE viral transcription factor) to vDNA and the onset of viral gene expression (Fig 6A–6D). Thus, we have identified a temporal boundary in the recruitment of intrinsic and innate immune regulators to infecting viral genomes that could represent a shift in host response; from an intrinsic defence centred on the repression of viral gene expression to the induction of innate immune signalling that promotes an antiviral state to restrict virus propagation. This hypothesis is supported by our observation that the induction of innate immunity requires the onset of vDNA replication (Fig 7E and 7I). IFI16 is reported to recognise nucleosome free DNA in a sequence independent manner [85–87], with high binding affinity for G quadraplex, branched or cruciform DNA structures [52]. Thus, it likely that the recruitment of IFI16 to infecting viral genomes is stabilised following the onset of vDNA replication that produces an abundance of such DNA structures [57]. This hypothesis may account for why PAA, but not ACG, is capable of inhibiting the induction of ISG expression (Fig 7E and 7F). As inactivation of the vDNA polymerase by PAA would be expected to impair the initiation of vDNA replication [53, 57], while ACG treatment would lead to the accumulation of stalled vDNA replication products [54]. Thus, we hypothesize that the topology of replicating vDNA is important for the stable recruitment of IFI16 on to vDNA that leads to the induction of ISG expression and type-I IFN production during herpesvirus infection. We note that other viral and cellular factors are likely to contribute to the induction of innate immunity under alternative infection conditions, for example excessively high MOI, UV inactivation, or the use of viral mutants defective in multiple genes, which may deliver or generate PAMPs for PRR detection at different stages of infection.

While it is clear that IFI16 plays a key role in the induction of ISG expression during herpesvirus infection, we have also identified an important and novel role for PML in this host response to HSV-1 infection (Fig 8). Depletion of PML significantly reduced the levels of ISG transcript accumulation observed at 9 hpi during HSV-1 ICP0-null mutant infection (Fig 8F), a time point which proceeds ISG expression (16 hpi; Fig 7B). Thus, under infection conditions that saturate intrinsic PML-NB host defences during HSV-1 ICP0-null mutant infection (MOI ≥ 1 PFU/cell, ~ 25 genome copies/cell), PML plays a significant role in mediating the induction of ISG transcription. Correspondingly, pharmacological inhibition of JAK signalling by Ruxolitinib [58, 59] did not enhance HSV-1 ICP0-null mutant propagation in either IFI16 or PML depleted cells (Fig 8D). Collectively, these data demonstrate that PML plays an important role in the induction of innate immunity in response to HSV-1 infection that restricts the propagation of HSV-1 following the successful saturation of PML-NB intrinsic host defences. These observations are consistent with reports highlighting a role for PML to mediate the induction of innate immunity in response to other human herpesviruses [88–90], and a growing body of literature suggesting that specific PML isoforms play an important role in mediating the transcriptional regulation of cytokine signalling (reviewed in [91]). Notably, PML has been reported to mediate the recruitment of activated STAT1 and 2, along with HDAC1 and 2, onto ISG promoters (*ISG54*, *CXCL10*) during human cytomegalovirus (HCMV) infection [90]. This host response is antagonized by the HCMV IE gene product IE1 [88, 90], a viral protein known to disrupt PML-NBs and to relieve the intrinsic cellular restriction of an HSV-1 ICP0-null mutant [78]. As JAK activity is well known to be required for STAT phosphorylation [60], these observations are consistent with our inhibitor studies (Figs 7H, 7I, 8B and 8D), which show JAK activity to play an important role in the induction of ISGs during HSV-1 ICP0-null mutant vDNA replication at 9 hpi. Consistent with STAT1 depletion studies [61], JAK inhibition did not influence the intrinsic restriction of an HSV-1 ICP0-null mutant (Fig 8A). These data contrast with depletion studies, which show a clear cooperative role for PML-NB host factors to restrict the initiation of HSV-1 ICP0-null replication [19, 50, 69]. Taken together with our observations in RPE cells (Figs 9A and 10), which are restrictive to ICP0-null HSV-1 replication but defective in innate immune signalling, these data show that PML plays dual roles in the temporal regulation of both intrinsic and innate immunity in response to HSV-1 infection. Host defences that are counteracted by ICP0 through the degradation of PML and disruption of PML-NBs from the outset of infection.

In conclusion, we have shown for the first time that the differential recruitment of host immune regulators to infecting viral genomes plays a fundamental role in the sequential regulation of intrinsic and innate immune defences following HSV-1 nuclear infection. We have identified dual roles for PML in the regulation of these intracellular defences to HSV-1 infection that are dependent on vDNA entry into the nucleus and the onset of vDNA replication, respectively. Our analysis reconciles many long-standing questions as to the importance of PML and PML-NBs in the regulation of intracellular host immunity during HSV-1 infection. Our data highlights the importance of viral antagonists that disrupt PML-NBs to inactivate and evade key intracellular immune defences from the outset of infection, thereby promoting the onset of replication, propagation, and ultimately transmission to new hosts. Moreover, we demonstrate the versatility and sensitivity of bio-orthogonal labelling of viral nucleic acid to investigate the temporal recruitment of host immune regulators to infecting viral genomes during infection.

## Materials and methods

### Cells and drugs

Primary human foreskin fibroblast cells (HFs) were obtained from Thomas Stamminger (Department of Urology, University of Erlangen; [49]) and immortalized (HFt) by retrovirus transduction to express the catalytic subunit of human telomerase, as previously described [18]. HFt, retinal pigmented epithelial (RPE-1; ATCC, CRL-4000), Human osteosarcoma (U2OS and SAOS; ECACC, 92022711 and 89050205), primary human foetal lung fibroblast (MRC-5; ATCC, CCL-171), and adult human keratinocyte (HaCat; a gift of F. Rixon, MRC-UoG CVR) cells were grown in Dulbecco’s Modified Eagle Medium (DMEM; Life Technologies, 41966). HFt cells were cultured in the presence of 5 μg/ml of Hygromycin (Invitrogen, 10687–010) to maintain hTERT expression. Transduced HFt cells expressing shRNAs were cultured in the presence of Puromycin (Sigma-Aldrich, P8833; 1 μg/ml or 0.5 μg/ml for selection or maintenance, respectively). Primary human embryonic lung fibroblast (HEL 299; ECACC, 87042207) cells were maintained in Minimum Essential Medium Eagle (MEM; Sigma-Aldrich M5650) supplemented with 2 mM L-Glutamine (Life Technologies, 25030–024) and 1 mM Sodium Pyruvate (Life Technologies, 11360–039). Baby hamster kidney fibroblast (BHK-21 C13; a gift of R. Everett, MRC-UoG CVR) cells were grown in Glasgow Minimum Essential Medium (GMEM; Life Technologies, 21710–025) supplemented with 10% Tryptose Phosphate Broth (TPB; Life Technologies, 18050–039). Medium for all cell lines was supplemented with 10% foetal bovine serum (FBS; Life Technologies, 10270), 100 units/ml penicillin, and 100 μg/ml streptomycin (Life Technologies, 15140–122). All cell lines were maintained at 37°C in 5% CO_2_. 5-Ethynyl-2’-deoxyuridine (EdU; Sigma-Aldrich, T511285), 5-Ethynyl-2’-deoxycytidine (EdC; Sigma-Aldrich, T511307), 2’-deoxyruridine (dU; Sigma-Aldrich, D5412), and Ruxolitinib (Ruxo; Sellechchem, S1378) were prepared in DMSO and used at the indicated concentrations. Acycloguanosine (ACG, Sigma-Aldrich, A4669), Phosphonoacetic acid (PAA, Sigma-Aldrich, 284270), and Interferon beta (IFN-β; Calbiochem, 407318) were prepared in Milli-Q H_2_O and used at the indicated concentrations.

### Viruses

Wild-type HSV-1 strain 17syn+ (HSV-1), its ICP0-null mutant derivative *dl*1403 (ΔICP0; [33]), and their respective variants that express eYFP.ICP4 [40] were propagated and titrated as described [35]. For EdU labelling of viral genomes, RPE cells were infected with either HSV-1 (MOI 0.001 PFU/cell) or ΔICP0 (MOI 0.5 PFU/cell). At 24 h post-infection (hpi), EdU or EdC was added at a final concentration of 0.5 μM, unless otherwise indicated. Fresh EdU/EdC was pulsed into infected cultures at 24 h intervals until extensive cytopathic effect was observed, typically 3 to 4 days post-infection. Supernatants containing labelled cell released virus (CRV) were clarified by centrifugation (423 x*g* for 10 min) and filtered through a 0.45 μm sterile filter and pelleted using a Beckman TLA100 Ultracentrifuge (33,800 x*g* for 3h at 4°C). Virion pellets were resuspended and pooled in 500 μl complete DMEM medium, and titrated in U2OS cells as described [35].

### Plasmids and lentiviral transduction

Plasmids encoding short hairpin (sh) RNAs against a non-targeted control sequence (shCtrl; 5’-TTATCGCGCATATCACGCG-3’), PML (shPML; 5’-AGATGCAGCTGTATCCAAG-3’), ATRX (shATRX; 5’- CGACAGAAACTAACCCTGTAA-3’), or IFI16 (shIFI16; 5’-CCACAATCTACGAAATTCA-3’) were used to generate lentiviral supernatant stocks for transduction of HFt cells as described [11, 49, 65]. Pooled and stably transduced cells were used for experimentation.

### Antibodies

The following antibodies were used for immunofluorescence or western blotting: Primary rabbit polyclonal: anti-actin (Sigma-Aldrich, A5060), anti-Daxx (Upstate, 07–471), anti-ATRX (Santa Cruz, H300), anti-PML (Bethyl Laboratories, A301-167A; Jena Biosciences, ABD-030), anti-Sp100 (GeneTex, GTX131569), anti-SUMO2/3 (Abcam, ab22654), anti-Mx1 (Santa Cruz, sc-50509;ProteinTech, 13750-1-AP), anti-ISG15 (ProteinTech, 15981-1-AP), and anti-ISG54 (IFIT2, proteinTech, 12604-1-AP). Primary mouse monoclonal: anti-ICP0 (11060, [92]), anti-ICP4 (58s, [93]), anti-VP5 (DM165, [94]), anti-SUMO2/3 (Abcam, ab81371), anti-PML (abcam, ab96051), anti-IFI16 (abcam, ab55328; Santa Cruz, sc-8023). Primary antibodies were detected using the following secondary antibodies: DyLight-680 or -800 conjugated anti-rabbit or -mouse (Thermo; 35568 and SA5-35571), Alexa -488, -555, or -647 conjugated anti-rabbit, or -mouse (Invitrogen; A21206, A21202, A31572, A31570, A31573, A31571), HRP conjugated anti-mouse (Sigma-Aldrich, A4416).

### Plaque Forming Efficiency (PFE)

Unless otherwise stated, cells were infected with serial dilutions of HSV-1 or ΔICP0 and rocked every 10 min for 1 h prior to overlay with medium supplemented with 2% Human Serum (HS; MP Biomedicals, 2931149). 24 to 36 hpi, cells were washed twice in PBS (Sigma-Aldrich, D1408), simultaneously fixed and permeabilized in 1.8% formaldehyde (Sigma-Aldrich, F8775) and 0.5% NP40 (BDH, 56009) in PBS for 10 min, then washed twice in 0.1% Tween in PBS (PBST). Cells were blocked with 5% skimmed milk powder (SMP; Marvel) in PBST (blocking buffer) for 30 min before incubation with an anti-VP5 monoclonal antibody diluted in blocking buffer for 90 min. Cells were washed three times with PBST, incubated with HRP conjugated anti-mouse IgG diluted in blocking buffer for 60 min, then washed with PBST three times. Plaques were visualized with True Blue peroxidase developing solution (Insight, 50-78-02) according to the manufacturer’s instructions, and washed with Milli-Q H_2_O prior to plaque counting or imaging using an Axio Observer Z.1 microscope (Zeiss) with differential interference contrast. For plaque formation efficiency (PFE) assays, plaque counts are expressed relative to the number of plaques on control HFt or U2OS infected cell monolayers (as indicated) at the equivalent dilution of input virus. Results presented as relative fold change (number of plaques sample/number of plaques sample control). Plaque diameters were measured using Zen blue (Zeiss) imaging software.

### Viral yield assays

Cells were infected with either HSV-1 (MOI 0.001 PFU/cell), or ΔICP0 (MOI 1 or 2 PFU/cell, as indicated) and rocked every 10 min for 1 h prior to overlay with complete medium containing either 5 μM Ruxolitinib or DMSO as a carrier control. Supernatants containing cell released virus (CRV) were collected at the indicated times post-infection. Virus titres were calculated by titration on U2OS cells as described [35].

### Particle counting

Equal volumes of virus suspension and polystyrene latex spheres (Agar Scientific, AGS130-02) at a known concentration per ml were mixed in 2 volumes of TNE buffer (20 mM Tris [pH 7.5], 0.5 M NaCl, and 1 mM EDTA). 5 μl of suspension was then added to a glow discharged EM grid (Agar Scientific, S162-4), allowed to rest for 1 min, washed three times in deionised water, and stained with Ammonium Molybdate (2% (w/v) pH 7.2). Dry grids were examined using a JEM2200 FS electron microscope (JEOL) and images captured using an Ultrascan 4000 charge-coupled-device (CCD) camera (Gatan). Multiple images (n ≥ 6) per sample were used for virus particle and latex bead enumeration and used to calculate the number of particles per ml of virus stock inoculum.

### Western blotting

Treated or infected cells were washed twice with PBS. Whole cell lysates were collected in SDS-PAGE loading buffer containing 4 M Urea (Sigma-Aldrich, U0631) and 50 mM Dithiothreitol (DTT; Sigma-Aldrich, D0632). Proteins were resolved on NuPAGE 4–12% Bis-Tris Protein gels (Invitrogen, NP0322BOX) in MES (Invitrogen; NP0002) or MOPS buffer (Invitrogen, NP0001) and transferred onto 0.2 μm nitrocellulose membrane (Amersham, 15249794) for 90 min at 30 volts in Novex transfer buffer (Invitrogen, NP0006-1) according to the manufacturer’s instructions. Membranes were blocked in PBS with 5% FBS (Block) for a minimum of 1 h at room temperature. Membranes were incubated in primary antibody diluted in Block for a minimum of 1 h, washed three times with PBST for 5 min each, then incubated in secondary antibody diluted in Block for 1 h. Following three 5 min washes in PBST, one 5 min wash in PBS, and one rinse in Milli-Q H_2_O, membranes were imaged on an Odyssey Infrared Imager (LiCor). The intensity of protein bands was quantified with Odyssey Image Studio Software.

### Immunofluorescence and confocal microscopy

Cells were seeded overnight on to 13 mm coverslips prior to treatment or infection at the indicated MOI and time points at 37°C. For click chemistry assays, cells were washed in serum free DMEM prior to overlay in complete medium or fixation. At indicated time points, cells were washed twice in CSK buffer (10 mM HEPES, 100 mM NaCl, 300 mM Sucrose, 3 mM MgCl_2_, 5 mM EGTA), simultaneously fixed and permeabilized in 1.8% formaldehyde and 0.5% Triton-X100 (Sigma-Aldrich, T-9284) in CSK buffer for 10 min, and washed twice in CSK. Coverslips were then blocked with 2% HS in PBS for 30 min prior to click chemistry followed by immunostaining. Where applicable, EdU-labelled vDNA was detected using the Click-iT Plus EdU Alexa Fluor 555 Imaging Kit (ThermoFisher scientific, C10638) according to the manufacturer’s instructions. For host and viral protein labelling, cells were incubated with primary antibodies diluted in 2% HS in PBS for 60 min, then washed in PBS three times, before incubation with secondary antibodies and DAPI (Sigma-Aldrich, D9542) in 2% HS in PBS for 60 min. Coverslips were then washed in PBS three times, and twice in Milli-Q H_2_O prior to mounting on Citiflour AF1 (Agar Scientific, R1320). Coverslips were examined using a Zeiss LSM 880 confocal microscope using the 63x Plan-Apochromat oil immersion lens (numerical aperture 1.4) using 405 nm, 488 nm, 543 nm, 594 nm, and 633 nm laser lines. Zen black software (Zeiss) was used for image capture, generating cut mask channels, and calculating weighted colocalization coefficients. High-resolution Z-series images were captured under LSM 880 Airy scan deconvolution settings using 1:1:1 capture conditions at 0.035 μm intervals. Images were processed using Imaris (Bitplane) software to produce rendered 3D image reconstructions and to calculate Pearson colocalization coefficients. Exported images were processed with minimal adjustment using Adobe Photoshop and assembled for presentation using Adobe Illustrator.

### Virion genome release assay

*In vitro* virion DNA release assays were conducted as essentially described in [47]. Briefly, 1x10^8^ PFU of virus preparation was diluted in ice-cold TNE buffer in the presence or absence of GuHCl (2M final concentration; Sigma-Aldrich, G3272). Samples were incubated on ice for 60 mins prior to the addition of ice-cold Methanol (final concentration 60%). Samples were dried onto poly-D-lysine (Sigma-Aldrich, P7405) treated coverslips for 60–90 mins at 4°C prior to fixation in PBS containing 1.8% formaldehyde and 0.5% Triton-X100 for 10 mins at RT. Samples were washed twice in PBS and blocked in PBS containing 2% FBS for 10 mins at RT. Samples were processed for click chemistry to detect EdU or EdC labelled vDNA and immuno-stained for VP5 to detect viral capsids (as described above). High-resolution Z-series images were captured under LSM 880 Airy scan deconvolution settings at 0.2 μm intervals (as described above) and the number of capsids and EdU or EdC labelled viral genomes were quantified in Zen blue (Zeiss) from maximum intensity projection images.

### Plaque-edge recruitment assays

HFt cells were infected with ΔICP0 EYFP.ICP4 at an MOI of 2 PFU/cell to enable the initiation of viral replication and plaque formation, as previously described [9]. At 24 hpi, infected cell monolayers were pulsed with 1 μM EdU for 6h prior to fixation and immunostaining, as described above.

### Quantitative RT-PCR

Cells were mock, HSV-1, or ΔICP0-infected at the indicated MOI, and total RNA collected at 9 hrs post-infection unless otherwise stated. Where applicable, all drug treatments were added at the indicated concentration 1 h after inoculum adsorption. Total RNA was isolated using the RNAeasy Plus Kit (Qiagen, 74134) according to the manufacturer’s instructions. Reverse transcription (RT) was performed using the TaqMan Reverse Transcription Reagents kit (Life Technologies, N8080234) with oligo(dT) primers. cDNA samples were analyzed in triplicate using TaqMan Fast Universal PCR Master Mix (Life Technologies, 4352042) with the following TaqMan gene specific primer-(FAM/MGB) probe mixes (Life Technologies): PML (assay ID Hs00231241_m1), IFI16 (assay ID Hs00986757_m1), ATRX (assay ID HS00997529_m1), Mx1 (assay ID HS00895608_m1) ISG15 (assay ID HS01921425_s1), ISG54 (assay ID Hs01922738_s1), or GAPDH (4333764F) on a 7500 Fast Real time PCR system (Applied Biosystems). Relative mRNA levels were determined using the ΔΔCt method, normalized to GAPDH, and expressed relative to indicated treatments. Data presented is from a minimum of two independent biological replicates, each analysed in triplicate (RQ/RQmin/max). Means (RQ) and standard deviations (RQmin/max) are presented. For input viral genome quantitation, vDNA was extracted from infected HFt cells harvested at 90 mpi. Cells were trypsinised, pelleted by centrifugation (500 x*g*, 10 min), washed twice in PBS, and resuspended in PBS containing 1% SDS and 300mM Sodium acetate (pH 5.2). Total DNA was isolated by phenol chloroform extraction and ethanol precipitation, and resuspended in Tris buffer (10mM Tris-HCl pH 8.5). qPCR was performed using two virus specific (UL30 and UL36) primer-probe sets with distinct fluorophores (Sigma-Aldrich; S3 Table) in duplex reactions performed in triplicate per biological replicate. Quantitation was performed against standards of known concentration derived from a purified infectious HSV-1 17syn+ BAC clone (SR27 DNA, [95]; a kind gift from Andrew Davison MRC-UoG CVR).

## Supporting information

S1 Fig HSV-1 replication is sensitive to EdU or EdC labelling in a cell type and ICP0-dependent manner. (A, D, G) RPE or HEL cells (as indicated) were infected with 100 PFU of either WT or ΔICP0 HSV-1 and incubated in the presence of DMSO, ACG (50 μM), EdU or EdC (0.5–10 μM, as indicated) for 24 h. Plaque counts were determined and expressed relative to DMSO control (1) and presented as relative PFE. n ≥ 3, means and standard deviations shown. (B, E, H) RPE or HEL cells (as indicated) were infected (as in A) and plaque diameters measured at 24 hpi. Boxes: 25^th^ to 75^th^ percentile range; black line: median plaque size; whiskers: 5^th^ to 95^th^ percentile range. n ≥ 100 plaque measurements from 4 independent infections. (C, F, I) RPE or HEL cells (as indicated) were infected with either WT (MOI 0.001 PFU/cell) or ΔICP0 (MOI 2 PFU/cell) HSV-1 in the presence of EdU or dU at the indicate concentrations. CRV was collected at 48 hpi and titres determined on U2OS cells. n ≥ 3, means and standard deviations are shown.
(EPS)

S2 Fig Detection of viral genomes within HSV-1^EdC^ virions requires permeabilization of the capsid by GuHCl treatment. 1x10^8^ PFU of HSV-1^EdC^ virions were incubated in TNE buffer or TNE buffer containing 2M GuHCl at 4°C for 60 mins, as described in [47]. EdC labelled vDNA (red) and capsids (green) were detected by click chemistry and indirect immunofluorescence staining for VP5 (the major capsid protein), respectively.
(EPS)

S3 Fig PML-NB proteins entrap vDNA upon nuclear entry. Individual channel images for data presented in Fig 3. Localization of PML (green) with HSV-1^EdU^ vDNA (red, white arrows), and PML-NB constituent proteins (Daxx, Sp100, ATRX, SUMO2/3) or IFI16 (cyan, as indicated) at 90 mpi (post-addition of virus; MOI of 3 PFU/cell) or equivalent mock infected cells (as indicated). Insets show magnified regions of interest (dashed boxes) highlighting host protein localization with vDNA. Cut mask (yellow) highlights regions of colocalization between host proteins and vDNA (as indicated). Weighted colocalization coefficients are shown.
(EPS)

S4 Fig PML-NBs entrap HSV-1 vDNA in an ICP0-independent manner. Confocal microscopy images as for data presented in Fig 3 for ΔICP0^EdU^ infection. Localization of PML (green) with infecting ΔICP0^EdU^ vDNA (red, white arrows) and PML-NB constituent proteins (Daxx, Sp100, ATRX, SUMO2/3) or IFI16 (cyan, as indicated) at 90 mpi (post-addition of virus; MOI of 3 PFU/cell). Insets show magnified regions of interest (dashed boxes) highlighting host protein localization with vDNA. Cut mask (yellow) highlights regions of colocalization between host proteins and vDNA (as indicated). Weighted colocalization coefficients are shown.
(EPS)

S5 Fig IFI16 and PML colocalization with vDNA over a range of MOI. (A,B) HFt cells were infected with HSV-1^EdU^ over a range of MOIs (1–50 PFU/cell, as indicated). Cells were fixed and permeabilized at 90 mpi (post-addition of virus). vDNA, IFI16 and PML, were detected by click chemistry and indirect immunofluorescence staining, respectively. (A) Confocal microscopy images showing IFI16 (green) dots at the nuclear rim in association with PML (cyan) and vDNA (red) at an MOI of 50. White arrow highlights vDNA colocalization with IFI16 and PML. Yellow arrow highlights vDNA colocalization with PML only. Correspondingly coloured insets show magnified regions of interest (dashed boxes). Cut mask (yellow) highlights regions of colocalization between IFI16, PML, and vDNA (as indicated). Weighted (w.) colocalization coefficients shown. (B) Scatter plot showing paired w. colocalization coefficients of IFI16 and PML to individual nuclear infecting viral genomes (as described above). n ≥ 250 genomes per sample population derived from a minimum of two independent infections. (C) Quantitation of host protein recruitment to infecting viral genomes (as in B). Boxes: 25^th^ to 75^th^ percentile range; black line: median weighted (w.) colocalization coefficient; whiskers: 5^th^ to 95^th^ percentile range. Solid line indicates coincident threshold level (weighted colocalization coefficients < 0.2). (D) HFt cells were HSV-1^EdU^ infected at an MOI 10 PFU/cell. Cells were fixed and permeabilized at either 15 or 30 mpi (post-addition of virus). Scatter plot showing paired w. colocalization coefficients of IFI16 and PML to individual nuclear infecting viral genomes. n ≥ 60 genomes per sample population derived from a minimum of two independent infections. (E) Quantitation of host protein recruitment to infecting viral genomes (as shown in D). Boxes: 25^th^ to 75^th^ percentile range; black line: median weighted (w.) colocalization coefficient; whiskers: 5^th^ to 95^th^ percentile range. Solid line indicates coincident threshold level (weighted colocalization coefficients < 0.2). ** *P* < 0.01, *** *P* < 0.001, ns (not significant); Mann-Whitney *U*-test.
(EPS)

S6 Fig Depletion of PML does not enhance IFI16 recruitment to ΔICP0^EdU^ infecting viral genomes. Individual channel images for data presented in Fig 4 for ΔICP0^EdU^ infection. Localization of PML (green), and either Daxx or IFI16 (cyan; as indicated) to infecting ΔICP0^EdU^ vDNA (red, white arrows) in HFt shCtrl and shPML cells at 90 mpi (post-addition of virus; MOI of 3 PFU/cell). Insets show magnified regions of interest (dashed boxes) highlighting host protein localization with vDNA. Cut mask (yellow) highlights regions of colocalization between PML, IFI16, Daxx, and vDNA (as indicated). Weighted colocalization coefficients are shown.
(EPS)

S7 Fig Recruitment of IFI16 and eYFP.ICP4 to ΔICP0^EdU^ vDNA in an asynchronous plaque-edge recruitment assay. HFt cells were infected with ΔICP0.eYFP.ICP4 at an MOI of 2 PFU/cell for 24h prior to pulse labelling with EdU for 6h. Representative images show the cellular localization of eYFP.ICP4 (green), vDNA (red), and IFI16 (cyan) in cells associated with a developing ΔICP0.eYFP.ICP4 plaque. (Left) Wide-field view of the plaque-body with newly infected cells on the periphery of the plaque-edge highlighted (dashed boxes; regions of interest 1–3). (Right) Single cell images of regions of interest (dashed boxes 1–3, respectively) showing nuclei at different stages of infection. Box 1: Infected nucleus with robust vDNA replication compartments. Box 2: Asymmetrically infected nucleus with early stage vDNA replication compartments. Box 3: Asymmetrically distributed EdU labelled nuclear infecting viral genomes that have yet to initiate viral IE (eYFP.ICP4) gene expression. IFI16 is only detected in association with vDNA that has initiated IE gene expression.
(EPS)

S8 Fig PML-NB entrapment of vDNA occurs in a cell type dependent manner. Individual channel images from data presented in Fig 10. Localization of PML (green) and Daxx (cyan) with vDNA (red, white arrows) in HSV-1^EdU^ infected HFt, RPE, U2OS, or SAOS cells at 30 mpi (post-addition of virus; MOI 3 PFU/cell). Insets show magnified regions of interest (dashed boxes) highlighting PML and Daxx localization with vDNA. Cut mask (yellow) highlights regions of colocalization between PML or Daxx and vDNA (as indicated). Weighted colocalization coefficients are shown.
(EPS)

S1 Table EdU or EdC treatment of infected cell monolayers inhibits wild-type (strain 17syn+) and ICP0-null mutant (*dl*1403/ΔICP0) HSV-1 plaque formation efficiency (PFE) in a cell-type and dose-dependent manner. Plaque counts expressed relative to DMSO control monolayers, (# of plaques treated / # of plaques DMSO control) at equivalent serial dilutions of virus and presented as relative plaque formation efficiency (PFE). n ≥ 3, means and standard deviations (in brackets) shown. Small plaque phenotypes at 24–36 hpi highlighted.
(DOCX)

S2 Table Particle to PFU and genome to PFU ratios of EdU labelled wild-type and ICP0-null mutant (*dl*1403/ΔICP0) HSV-1 stock preparations in permissive (U2OS) and restrictive (HFt) cell types. Mean particles/ml determined from a minimum of 6 independent fields of view. Mean PFU/ml determined from 3 independent titrations on either U2OS or HFt (as indicated). Mean genome copy number/U2OS PFU determined from triplicate qPCR reactions from 2 independent experiments. *PS*, plate stock (no ultracentrifugation).
(DOCX)

S3 Table Primer-probe sets used for HSV-1 vDNA quantitation by qPCR. (DOCX)
