# Correction: Distinct temporal roles for the promyelocytic leukaemia (PML) protein in the sequential regulation of intracellular host immunity to HSV-1 infection

**DOI:** 10.1371/journal.ppat.1006927

**Published:** 2018-02-22

**Authors:** Thamir Alandijany, Ashley P. E. Roberts, Kristen L. Conn, Colin Loney, Steven McFarlane, Anne Orr, Chris Boutell

In the Funding section, the grant number from the funder Medical Research Council (MRC) is listed incorrectly. The correct grant number is: MC_UU_12014/5.

## References

[1] Alandijany T, Roberts APE, Conn KL, Loney C, McFarlane S, Orr A, et al (2018) Distinct temporal roles for the promyelocytic leukaemia (PML) protein in the sequential regulation of intracellular host immunity to HSV-1 infection. PLoS Pathog 14(1): e1006769 https://doi.org/10.1371/journal.ppat.1006769 29309427 10.1371/journal.ppat.1006769 PMC5757968

