# Supplementary material for: Distinct temporal roles for the promyelocytic leukaemia (PML) protein in the sequential regulation of intracellular host immunity to HSV-1 infection
Source: PLoS Pathog. 2018 Jan 8;14(1):e1006769. doi: 10.1371/journal.ppat.1006769 (PMC5757968; doi:10.1371/journal.ppat.1006769)
Supplement: S1 Table — Plaque counts expressed relative to DMSO control monolayers, (# of plaques treated / # of plaques DMSO control) at equivalent serial dilutions of virus and presented as relative plaque formation efficiency (PFE). n ≥ 3, means and standard deviations (in brackets) shown. Small plaque phenotypes at 24–36 hpi highlighted. (DOCX) [file ppat.1006769.s009.docx]

| Cell line  (Virus) | Type (species/organ) | DMSO | ACG  (µM) | EdU  (µM) | | | |  | EdC  (µM) | | | | Small plaque phenotype (yes/no) |
| --- | --- | --- | --- | --- | --- | --- | --- | --- | --- | --- | --- | --- | --- |
|  |  |  | 50 | 0.5 | 1.0 | 5.0 | 10 |  | 0.5 | 1.0 | 5.0 | 10 |  |
| BHK-21 C13  (17+) | Fibroblast (hamster/kidney) | 1.0 | 0 | 0.83 (0.19) | 0.81 (0.19) | 0.45 (0.21) | 0.24 (0.11) |  | 0.70 (0.22) | 0.68 (0.22) | 0.38 (0.16) | 0.19 (0.07) | yes |
| HEL 299  (17+) | Fibroblast (human/lung) | 1.0 | 0 | 0.89 (0.13) | 0.95 (0.12) | 0.34 (0.27) | 0.07 (0.04) |  | 1.03 (0.26) | 0.72 (0.08) | 0.22 (0.25) | 0.01 (0.03) | yes |
| MRC5t  (17+) | Fibroblast (human/lung) | 1.0 | 0 | 1.11 (0.18) | 0.94 (0.24) | 0.29 (0.05) | 0.00 (0.00) |  | 0.85 (0.22) | 0.83 (0.09) | 0.1 (0.11) | 0.00 (0.00) | yes |
| HFt  (17+) | Fibroblast (human/foreskin) | 1.0 | 0 | 1.04 (0.14) | 1.04 (0.22) | 1.0 (0.33) | 0.66 (0.39) |  | 0.96 (0.15) | 0.76 (0.22) | 0.54 (0.21) | 0.12 (0.07) | yes |
| HaCat  (17+) | Keratinocyte  (human/skin) | 1.0 | 0 | 1.03 (0.06) | 1.11 (0.12) | 1.15 (0.14) | 0.77 (0.14) |  | 1.09 (0.07) | 1.12 (0.11) | 1.04 (0.15) | 0.81 (0.21) | no |
| RPE  (17+) | Epithelial  (human/retina) | 1.0 | 0.57 (0.13) | 1.03 (0.07) | 1.03 (0.08) | 1.04 (0.12) | 0.91 (0.08) |  | 0.96 (0.07) | 0.96 (0.10) | 0.88 (0.08) | 0.95 (0.10) | no |
| U2OS  (17+) | Epithelial  (human/bone osteosarcoma) | 1.0 | 0 | 0.43 (0.14) | 0.33 (0.07) | 0.07 (0.07) | 0.01 (0.02) |  | 0.34 (0.06) | 0.18 (0.07) | 0.01 (0.01) | 0 | yes |
| SAOS  (17+) | Epithelial  (human/bone osteosarcoma) | 1.0 | 0.06 (0.00) | 0.38 (0.12) | 0.37 (0.09) | 0.15 (0.14) | 0.05 (0.06) |  | 0.40 (0.20) | 0.38 (0.15) | 0.22 (0.15) | 0.12 (0.11) | yes |
| HEL 299  (*dl*1403) | Fibroblast (human/lung) | 1.0 | 0 | 0.02 (0.02) | 0 | 0 | 0 |  | 0.03 (0.04) | 0.03 (0.04) | 0 | 0 | yes |
| RPE  (*dl*1403) | Epithelial  (human/retina) | 1.0 | 0.02 (0.01) | 0.46 (0.07) | 0.43 (0.06) | 0.01 (0.00) | 0 |  | 0.43 (0.06) | 0.38 (0.09) | 0 | 0 | yes |
| U2OS  (*dl*1403) | Epithelial  (human/bone osteosarcoma) | 1.0 | 0 | 0.73 (0.22) | 0.15 (0.19) | 0 | 0 |  | 0.01 (0.01) | 0 | 0 | 0 | yes |
| SAOS  (*dl*1403) | Epithelial  (human/bone osteosarcoma) | 1.0 | 0.01 (0.00) | 0.19 (0.1) | 0.14 (0.03) | 0.10 (0.04) | 0.03 (0.02) |  | 0.14 (0.07) | 0.13 (0.06) | 0.06 (0.02) | 0.02 (0.01) | yes |
