# Supplementary material for: Distinct temporal roles for the promyelocytic leukaemia (PML) protein in the sequential regulation of intracellular host immunity to HSV-1 infection
Source: PLoS Pathog. 2018 Jan 8;14(1):e1006769. doi: 10.1371/journal.ppat.1006769 (PMC5757968; doi:10.1371/journal.ppat.1006769)
Supplement: S2 Table — Mean particles/ml determined from a minimum of 6 independent fields of view. Mean PFU/ml determined from 3 independent titrations on either U2OS or HFt (as indicated). Mean genome copy number/U2OS PFU determined from triplicate qPCR reactions from 2 independent experiments. PS, plate stock (no ultracentrifugation). (DOCX) [file ppat.1006769.s010.docx]

| Virus (µM EdU) | Particles/ml | PFU/ml  (U2OS) | PFU/ml  (HFt) | Particle/  PFU  (U2OS) | Particle/  PFU  (HFt) | Particle/  PFU ratio  (HFt/U2OS) | Mean genome copy number/U2OS PFU  (infected HFt 90 mpi) | Relative genome copy number/cell  (U2OS MOI 0.1 PFU/cell) |
| --- | --- | --- | --- | --- | --- | --- | --- | --- |
| WT HSV-1*^PS^* (0) | nd | 2.4x10^8^ | 5.2x10^8^ | nd | nd | nd | 41.3 | 4.3 |
| WT HSV-1 (0) | 3.8x10^11^ | 4.8 x10^9^ | 5.3 x10^9^ | 79 | 72 | 0.9 | 27.6 | 2.8 |
| WT HSV-1 #1 (0.5) | 4.7 x10^11^ | 2.1 x10^9^ | 2.9 x10^9^ | 223 | 162 | 0.7 | 41.6 | 4.2 |
| WT HSV-1 #2 (0.5) | 4.1 x10^11^ | 1.7 x10^9^ | 2.5 x10^9^ | 241 | 164 | 0.7 | 42.7 | 4.3 |
| WT HSV-1 #3 (0.5) | 3.5 x10^11^ | 2.3 x10^9^ | 2.9 x10^9^ | 152 | 120 | 0.8 | 47.6 | 4.8 |
| ∆ICP0 (0.5) | 2.8 x10^11^ | 1.3 x10^9^ | 1.2 x10^6^ | 215 | 233333 | 1085 | 26.1 | 2.6 |
