# Supplementary material for: Distinct temporal roles for the promyelocytic leukaemia (PML) protein in the sequential regulation of intracellular host immunity to HSV-1 infection
Source: PLoS Pathog. 2018 Jan 8;14(1):e1006769. doi: 10.1371/journal.ppat.1006769 (PMC5757968; doi:10.1371/journal.ppat.1006769)
Supplement: S3 Table — (DOCX) [file ppat.1006769.s011.docx]

| HSV-1 target gene | Forward (5’ to 3’) | Reverse (5’ to 3’) | Probe |
| --- | --- | --- | --- |
| UL30 | GTGATCGGCGAGTACTGCAT | GTCCGGCAGAATAAAGCCCT | (JOE)-CCGCACCATCTACGACGGCC-(BHQ1) |
| UL36 | AAGAGGTGACGCGCTTACAA | GTAACAGGCGCGGATCAGTA | (6FAM)-CTGTCGCGACGCTACGTGCA-(BHQ1) |
